# Effects of sublethal doses of clothianidin and/or *V. destructor* on honey bee (*Apis mellifera*) self-grooming behavior and associated gene expression

**DOI:** 10.1038/s41598-019-41365-0

**Published:** 2019-03-26

**Authors:** Nuria Morfin, Paul H. Goodwin, Greg. J. Hunt, Ernesto Guzman-Novoa

**Affiliations:** 1 School of Environmental Sciences, University of Guelph, 50 Stone Road East, Guelph, N1G 2W1 Ontario, Canada; 2 Department of Entomology, Purdue University, 901 W State St, West Lafayette, IN 47907 United States of America

## Abstract

Little is known about the combined effects of stressors on social immunity of honey bees (*Apis mellifera*) and related gene expression. The interaction between sublethal doses of a neurotoxin, clothianidin, and the ectoparasite, *Varroa destructor*, was examined by measuring differentially expressed genes (DEGs) in brains, deformed wing virus (DWV) and the proportion and intensity of self-grooming. Evidence for an interaction was observed between the stressors in a reduction in the proportion of intense groomers. Only the lowest dose of clothianidin alone reduced the proportion of self-groomers and increased DWV levels. *V. destructor* shared a higher proportion of DEGs with the combined stressors compared to clothianidin, indicating that the effects of *V. destructor* were more pervasive than those of clothianidin when they were combined. The number of up-regulated DEGs were reduced with the combined stressors compared to clothianidin alone, suggesting an interference with the impacts of clothianidin. Clothianidin and *V. destructor* affected DEGs from different biological pathways but shared impacts on pathways related to neurodegenerative disorders, like Alzheimer’s, which could be related to neurological dysfunction and may explain their negative impacts on grooming. This study shows that the combination of clothianidin and *V. destructor* resulted in a complex and non-additive interaction.

## Introduction

The interaction of insects with their environment, such as to control ectoparasites, involves the activation of neural mechanisms controlling a variety of behaviors^1^. In the case of honey bees, behavioral immunity has been reported for the control of ectoparasites, like *Acarapis woodi* and *Varroa destructor*^2^. This is important as ectoparasites can cause damage to their hosts by physically obstructing airways and causing paralysis in the case of *A. woodi*^3^ and feeding on fat body tissue and haemolymph causing weight loss, as well as injecting saliva causing immunosuppression in the case of *V. destructor*^4,5^. *V. destructor* can also transmit honey bee viruses, like deformed wing virus (DWV) that result in wing deformity, reduced development and impaired learning^4,6,7^.

For honey bees, two means of social immunity against parasites are hygienic and grooming behaviors. Hygienic behavior involves the identification of diseased or dead brood followed by their removal from the colony^8^, and grooming behavior involves the removal of parasites from bees’ bodies using their legs and mandibles^9^. Both behaviors have been associated with the removal of *V. destructor* within a colony^8,10^. Grooming behavior can be divided into social grooming (or allogrooming) between nest mates and self-grooming for individual bees^9^. Self-grooming can be an effective restraint of *V. destructor* populations^10,11^, but is affected by the environment and genetic effects^12–14^.

Activation of neural mechanisms in insects is affected by neurotoxins, which are often used as insecticides for the control of agricultural pests. Some examples of commonly used neurotoxins against insects are organophosphates, pyrethroids, carbamates and neonicotinoids^15^. Their mode of action varies. Lethal doses of organophosphates disrupt acetylcholinesterase (AchE) activity^16^, pyrethroids prevent closure of voltage gated sodium channels in the axonal membranes^17^, and neonicotinoids mimic the neurotransmitter acetylcholine (ACh) causing acetylcholine receptors (nAChRs) to open ion channels leading to exhaustion and death^18^. However, sub-lethal doses of neurotoxic insecticides can also be damaging^19,20^. For example, for neonicotinoids, sub-lethal doses of imidacloprid reduced memory retention and altered motor function in honey bees^21,22^ and thiamethoxam impaired learning and memory in bumblebees^23^. Honey bees can be exposed to multiple sub-lethal doses of pesticides by gathering nectar and/or pollen when foraging (reviewed in refences^24^ and^25^). While there is no controversy on the effect of acute exposures to lethal doses of insecticides, like neonicotinoids, to non-target insects like honey bees, there is controversy about the effects of chronic exposure to sublethal doses (reviewed in reference^25^).

There are few studies on the importance of neural activity on grooming behavior in honey bees^13,26,27^. Also, there is little information about how neural activity can be affected by compounds and ectoparasites and their impact on grooming behavior. Thus, a study on the effect of a neonicotinoid, clothianidin, and *V. destructor* on neural mechanisms was undertaken using self-grooming behavior as the marker of the neural response. In this study, three sublethal doses of clothianidin with or without *V. destructor* were examined for the frequency and intensity of self-grooming behavior, DWV levels and honey bee gene expression, in order to better understand how a ACh agonist can act alone or interact with a honey bee parasite to affect a mechanism of social immunity.

## Results

### Self-grooming behavior

Significant effects of treatments were found for the proportion of bees that self-groomed in any manner versus bees that did not groom (Chi^2^_(7)_ = 36.019, p < 0.003) (Table 1). A *post-hoc* analysis revealed that the only significant difference was with the exposure to 9 × 10^−4^ ng/µl clothianidin alone which had the lowest proportion of bees that self-groomed (p < 0.003; Table 1). Also, the *post-hoc* analysis showed that there were no effects on the proportion of bees that self-groomed in the groups parasitized by *V. destructor*, with or without clothianidin. These results indicate that the only factor linked to a decrease in the proportion of bees that groomed in any manner was the exposure to the lowest dose of clothianidin without *V. destructor* and no interaction between the two stressors for that parameter was found.

**Table 1:** Contingency table showing the number of bees that self-groomed in any manner or that did not self-groom, and the proportion of bees that self-groomed in any manner within 3 min after placing 20 mg of flour on their thoraces that had previously been treated with clothianidin (0 ng/µl, 9 × 10^−4^ ng/µl, 4.2 × 10^−3^ ng/µl and 1 × 10^−2^ ng/µl) and/or *V. destructor*. The asterisk indicates a significant reduction in the proportion of bees that self-groomed based on Chi^2^ analysis and adjusted residuals and p value of 0.0031.

Treatment	Number of bees that self-groomed	Number of bees that did not self-groom	Proportion of bees that self-groomed
0 ng/µl	149	10	0.94
9 × 10^−4^ ng/µl	126	37	0.77*
4.2 × 10^−3^ ng/µl	135	11	0.92
1 × 10^−2^ ng/µl	168	32	0.84
0 ng/µl + *V. destructor*	106	8	0.93
9 × 10^−4^ ng/µl + *V. destructor*	74	19	0.80
4.2 × 10^−3^ ng/µl + *V. destructor*	88	20	0.81
1 × 10^−2^ ng/µl + *V. destructor*	57	16	0.78

Significant effects of treatments were also found for the proportion of bees that self-groomed intensively versus not groomed or self-groomed lightly (Chi^2^_(7)_ = 48.85, p < 0.0002) (Table 2). *Post-hoc* analysis showed that at 4.2 × 10^−3^ ng/µl clothianidin there was significantly less intense groomers with *V. destructor* than without *V. destructor*. There was also a significant reduction in the proportion of intense groomers for bees treated with 1 × 10^−2^ ng/µl clothianidin both with or without *V. destructor*. The significant reduction in the proportion of intense groomers in bees treated with 4.2 × 10^−3^ ng/µl clothianidin only in combination with *V. destructor* indicates a possible interaction between the two stressors for that parameter.

**Table 2:** Contingency table showing the number of bees that self-groomed intensively or that self-groomed lightly, and the proportion of bees that self-groomed intensively within 3 min after placing 20 mg of flour on their thoraces that had previously been treated with clothianidin (0 ng/µl, 9 × 10^−4^ ng/µl, 4.2 × 10^−3^ ng/µl and 1 × 10^−2^ ng/µl) and/or *V. destructor*. The asterisks indicate a significant reduction in the proportion of bees that self-groomed intensively based on Chi^2^ analysis and adjusted residuals and p value of 0.00019.

Treatment	Number of bees that groomed intensively	Number of bees that groomed lightly	Proportion of bees that groomed intensively
0 ng/µl	108	41	0.72
9 × 10^−4^ ng/µl	69	57	0.55
4.2 × 10^−3^ ng/µl	77	58	0.57
1 × 10^−2^ ng/µl	65	103	0.39*
0 ng/µl + *V. destructor*	59	47	0.56
9 × 10^−4^ ng/µl + *V. destructor*	39	35	0.53
4.2 × 10^−3^ ng/µl + *V. destructor*	36	52	0.41*
1 × 10^−2^ ng/µl + *V. destructor*	21	36	0.37*

### DWV quantification

**Figure 1:**
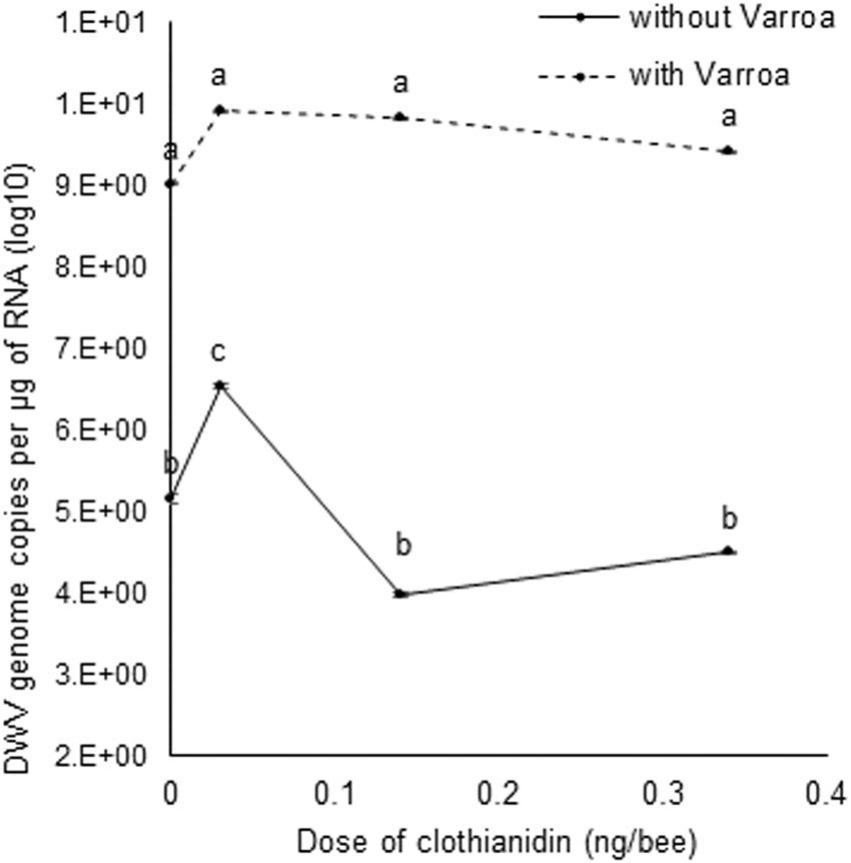
Mean DWV genome copies (GCs) per µg of RNA (±S.E.) of adult bees that were exposed to 0, 9 × 10^−4^, 4.2 × 10^−3^ and 1 × 10^−2^ ng of clothianidin per µl and/or *V. destructor*. Data points with different letters above them represent significant differences using Tukey’s HSD tests after a two-way ANOVA showed a significant effect.

Bees exposed to *V. destructor* had 1.8 log_10_ more DWV GCs per µg RNA than control bees (no clothianidin and no *V. destructor*) (F_(1,64)_ = 197.85, p =<0.0001) (Fig. 1). By comparison, bees exposed to 1 × 10^−4^ ng/µl clothianidin had 1.4 log_10_ more DWV GCs per µg RNA relative to the control (F_(3,64)_ = 3.84, p = 0.014). Thus, *V. destructor* parasitism has a much greater effect on DWV levels compared to the control than the lowest dose of clothianidin only. With treatments with higher doses of clothianidin, lower levels of DWV were observed in bees exposed to clothianidin alone compared to the lowest dose, whereas there was no significant change in DWV levels in bees exposed to both stressors compared to bees only parasitized with *V. destructor*. Therefore, the main factor contributing to DWV loads in the bees was *V. destructor* parasitism and the lowest dose of clothianidin without *V. destructor*.

### RNAseq

The number of reads per sample were 19,023,494 for 0 ng of clothianidin without *V. destructor*, 18,667,622 for 1 × 10^−2^ ng/µl of clothianidin without *V. destructor*, 16,164,788 for 0 ng of clothianidin plus *V. destructor* and 17,511,042 for 1 × 10^−2^ ng/µl of clothianidin plus *V. destructor*. Pairwise comparison between the control and 1 × 10^−2^ ng/ul clothianidin samples identified 267 up-regulated and 31 down-regulated DEGs (p < 0.05) showing the effects of clothianidin stressor alone included many more up-regulated than down-regulated DEGs (Supplementary Tables S1 and S2). Pairwise comparisons between the control and *V. destructor* alone samples had a similar number of up- and down-regulated genes (88 up-regulated and 78 down-regulated DEGs; p < 0.05) indicating similar numbers of up and down-regulated DEGs affected by *V. destructor* alone (Supplementary Tables S3 and S4). Pairwise comparison between the control and 1 × 10^−2^ ng/µl of clothianidin plus *V. destructor* samples revealed 62 up-regulated and 56 down-regulated DEGs (p < 0.05) again showing similar numbers of up and down-regulated DEGs affected by the combined stressors (Supplementary Tables S5 and S6).

The largest number of up-regulated DEGs occurred with clothianidin alone, but the average fold changes were quite similar for the DEGs with clothianidin alone (1.45) compared to *V. destructor* (1.32) and the combined stressors (1.38) (Supplementary Tables S1, S3 and S5). In contrast, the least number of down-regulated DEGs occurred with clothianidin alone, and the average log fold changes for the DEGs down-regulated by clothianidin alone (1.00) was also notably lower than that with *V. destructor* (1.90) and the combined stressors (1.57) (Supplementary Tables S2, S4 and S6).

### Up-regulated DEG pairwise comparisons

**Figure 2:**
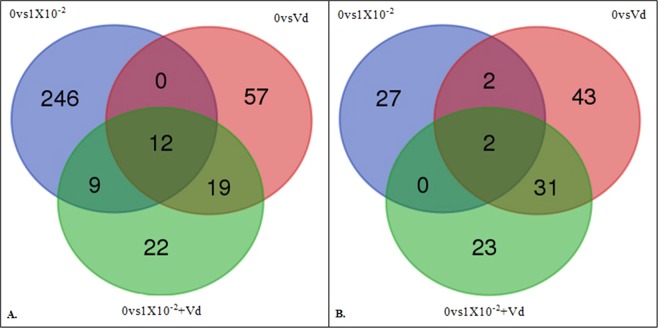
Venn diagram showing number of DEGs in the Differential Expression Analysis (DEA), and the genes in common between the pairwise comparisons of 0 ng of clothianidin vs 1 × 10^−2^ ng/µl of clothianidin (0vs1X10^−2^), 0 ng of clothianidin vs *V. destructor* (0vsVd) and 0 ng of clothianidin vs 1 × 10^−2^ ng/µl of clothianidin plus *V. destructor* (0vs1X10^−2^ + Vd). (**A**) Venn diagram showing the number of up-regulated DEGs (**B**). Venn diagram showing the number of down-regulated DEGs.

A Venn diagram of the up-regulated DEGs showed that 92.1% of the DEGs up-regulated by 1 × 10^−2^ ng/µl of clothianidin alone were unique to that treatment, none were shared solely with *V. destructor* alone, 3.4% were shared solely with the combined stressors, and 4.5% were shared among all the treatments (Fig. 2A and Supplementary Table S7). Thus, almost all those DEGs were unique to the effects of clothianidin. For the *V. destructor* alone treatment, 64.8% of the DEGs were not shared with the other treatments, none were shared only with clothianidin alone, 21.6% were shared only with the combined stressors and 13.6% were shared among all the pairwise comparisons. The majority of those DEGs were unique to *V. destructor* but many were shared, particularly with the combined stressor treatment. The combined stressors had just 35.5% of the DEGs being unique to that treatment, 14.5% shared only with clothianidin alone, 30.6% shared only with *V. destructor* alone and 19.4% shared among all the treatments. Thus, the combined stressors had the lowest number of DEGs unique to that treatment and was the only treatment where more DEGs were shared with other treatments (40) than were unique to that treatment (22).

Based on the number of up-regulated DEGs, it appears that the significantly up-regulated DEGs with the combination of *V. destructor* and clothianidin was more similar to that of *V. destructor* alone than to clothianidin alone. In contrast, there was relatively little in common among up or down-regulated DEGs between clothianidin alone and *V. destructor* alone showing that each stressor alone had different effects. The much smaller number of DEGs up-regulated by the combined stressors (62) compared to clothianidin alone (267) suggests that there was a possible interaction between clothianidin and *V. destructor* with the parasite reducing the number of DEGs when combined with clothianidin.

### Down-regulated DEG pairwise comparisons

A Venn diagram of the down-regulated DEGs showed that 87.1% of the DEGs down-regulated by 1 × 10^−2^ ng/µl of clothianidin alone were unique to that treatment, 6.5% were shared solely with *V. destructor* alone, none were shared solely with the combined stressors, and 6.5% were shared among all the libraries (Fig. 2B and Supplementary Table S7). Thus, like the up-regulated DEGs, almost all those DEGs were unique to the effects of clothianidin. For the *V. destructor* alone treatment, 55.1% of the DEGs were not shared with the other treatments, 2.6% were shared only with clothianidin alone, 39.7% were shared with the combined stressors and 2.6% were shared among all the treatments. Thus, a very similar number of those DEGs were shared between *V. destructor* and the combined stressor treatments. The combined stressors had just 41.07% of the DEGs being unique to that treatment, none were shared with clothianidin alone, 55.4% were shared only with *V. destructor* and 3.6% were shared among all the treatments. Like up-regulated DEGs, the combined stressors had the lowest number of DEGs unique to that treatment and was the only treatment where more DEGs were shared with other treatments (33) than were unique to that treatment (23).

Based on the number of down-regulated DEGs, similar conclusions as for up-regulated DEGs can be made about the greater similarity between the down-regulated DEGs of the combined stressors to that of *V. destructor* alone than to clothianidin alone. However, the number of DEGs down-regulated by the combined stressors (56) was not very different compared to clothianidin alone (31) as that observed for up-regulated DEGs, suggesting less possible interaction between clothianidin and *V. destructor*.

### KEGG analysis

KEGG pathway analysis was used to examine the link between DEGs and biological pathways and to compare which biological pathways were shared between treatments or unique to a particular treatment. KEGG analysis was able to assign approximately one quarter of all the up or down-regulated DEGs in this study to biological pathways (Supplementary Tables S8–S13). More than twice the number of KEGG pathways (256) were identified for up-regulated DEGs compared to down-regulated DEGs (106).

There were a number of noteworthy KEGG terms among the up-regulated DEGs (Supplementary Tables S8, S10 and S12). Some of the pathways among the 144 KEGG terms assigned to DEGs up-regulated by clothianidin that were unique to that treatment were associated with Parkinson’s disease. Among the 74 KEGG terms for up-regulated DEGs by *V. destructor*, viral myocarditis was notable among the pathways that were unique to *V. destructor*, and for the 27 KEGG terms for up-regulated DEGs with the combined stressors, notable KEGG terms unique to that treatment included insulin signaling pathway. For up-regulated KEGG pathways common between clothianidin and other treatments, noteworthy KEGG terms were Alzheimer’s disease, dopaminergic synapse and glutamatergic synapse shared with *V. destructor*, cellular senescence shared with the combined stressors, and calcium signalling pathway shared between all three treatments. For KEGG pathways common between *V. destructor* and the combined stressors, a noteworthy term the hippo signalling pathway-fly.

There were also a number of noteworthy KEGG terms among the down-regulated DEGs (Supplementary Tables S9, S11 and S13). For the 31 KEGG terms assigned to down-regulated DEGs by clothianidin, a notable term was sphingolipid metabolism. Among the 45 KEGG terms linked to down-regulated DEGs only by *V. destructor* were neuroactive ligand-receptor and among the 30 KEGG terms associated with the combined stressors was NOD-like receptor signaling pathway. For down-regulated KEGG pathways common between clothianidin and *V. destructor*, a possibly significant KEGG term was peroxisome, while longevity regulating pathway-multiple species was notable among the down-regulated DEGs shared by clothianidin, *V. destructor* and the combined stressors.

## Discussion

This study examined the effect of two stressors, sublethal doses of clothianidin and *V. destructor* on gene expression in the brain and increases in a mite-transmitted virus, DWV, to better understand how these two stressors act separately and together to affect honey bees on aspects of neural activity related to self-grooming behavior. Among the two stressors, clothianidin alone had a greater effect than *V. destructor* alone to reduce self-grooming behavior, based on the proportion of bees that self-groomed and the proportion of bees that self-groomed intensively, as well as the number of up-regulated DEGs. However, the results with the combination of *V. destructor* with clothianidin showed that combining each stressor did not result in a simple additive effect in the number of DEGs or the KEGG pathways associated with them.

One unexpected result of this study was that both DWV levels and the proportion of bees that self-groomed were affected only by the lowest dose of clothianidin without *V. destructor*. This may indicate that effects of DWV and grooming could be linked in some manner. Unexpectedly, higher doses of clothianidin in absence of mites, or the same dose of clothianidin combined with *V. destructor*, did not have the same impacts, indicating that there was a relatively specific impact from the lowest dose of clothianidin tested. Hormesis occurs when there is a beneficial biological response to a low exposure to a stressor^28^. In this case, a beneficial response did not occur at the lowest dose, but the effect could be similar to hormesis. At the lowest clothianidin dose, it would still bind to nicotinic acetylcholine receptors of the neural cells resulting in neural stimulation but may not overstimulate it to the level of toxic doses that block receptors impeding the action of the neurotransmitter ACh^29^. At very low doses, clothianidin stimulation of the central nervous system may just be sufficient to somehow interfere with self-grooming and resistance to a virus. While these results imply hormesis, future research should investigate more sublethal doses of clothianidin to confirm this by determining the range of doses that results in these effects.

While DWV levels were increased by the lowest dose of clothianidin without *V. destructor*, they were still much less than with *V. destructor* alone, which was not surprising as DWV can replicate in *V. destructor*, which also acts as a vector of DWV to bees^30^. An increase in DWV with the lowest dose of clothianidin without *V. destructor* indicates that the bees were latently infected with DWV, and the treatment permitted DWV to multiply in the bee. Latent infections of DWV in bees have been widely reported, and the levels of DWV in a bee may reflect the degree of resistance to the virus^31,32^. One explanation for increased DWV loads could be that the lowest dose of clothianidin suppressed humoral immunity in bees, which has been reported for a dose of clothianidin similar to the lowest dose in this study^33^. The mechanism proposed by Di Prisco *et al*.^33^ for this was that clothianidin negatively affected the NF-ƙβ signaling pathway, which regulates gene expression related to antiviral defence mechanisms^34^. However, another explanation could be that the neonicotinoid reduced hemocyte density, which occurred following sublethal doses of thiacloprid and imidacloprid in adult honey bees comparable to the lowest dose of clothianidin in this study^35^. Hemocytes are important for cellular defence mechanisms against viruses in insects through the phagocytosis of infected cells after induced apoptosis^36^. Higher levels of DWV infection could have contributed to impairment of the proportion of bees that groomed due to the virus affecting neural processes. Iqbal and Mueller^6^ showed that bees infected with DWV showed impairment of associative learning using the proboscis extension response (PER) assay. This could be due to DWV multiplication in the nervous system, including the brain^37^. Perhaps this is also true for DWV and grooming. Viral infections of insect brain tissues have been shown to affect behaviors, such as increased feeding activity in *Aedes aegypti* infected with dengue virus^38^.

Another explanation for the effects of the lowest dose of clothianidin on the expression of grooming behavior could be directly related to neural stimulation by clothianidin^29^. To perform self-grooming behavior, bees first have to perceive the stimulus of a substance, particle or pathogen on their bodies^39^. They then have to process that signal in the central nervous system and send the appropriate response through peripheral nerve conduction to the muscles^40^. Williamson *et al*.^22^ provided honey bees with sugar syrup containing even lower sublethal doses of clothianidin and thiamethoxam than in this study. After exposure, bees were not evaluated for the proportion that groomed, but for the time spent grooming, which was increased with the thiamethoxam but not with the clothianidin treatment. While the results are not directly comparable to this study, it does show that very low levels of a neonicotinoid can affect neural activity and grooming behavior.

Another parameter of grooming behavior, the intensity with which bees groom themselves, is important as it correlates well with the number of mites that they can remove from their bodies^10^. Thus far, there have been no studies of a neonicotinoid on this aspect of bee behavior. This study showed that at the medium dose, a significant reduction in the proportion of bees that groomed intensively occurred when exposed to both clothianidin and *V. destructor*, but not clothianidin alone, suggesting an interaction between the two stressors. This was not observed at the lowest and highest clothianidin doses. At the highest clothianidin dose, neural stimulation appears to be sufficient to affect neural activity associated to perceiving irritants on the bee’s body and reacting to them; including *V. destructor* did not increase that. In contrast, the lowest clothianidin dose apparently provided insufficient neural stimulation to affect intense grooming, even if *V. destructor* was included. Treatment with *V. destructor* alone was also insufficient to affect grooming intensity, despite the expected effect on energy stress by feeding on hemolymph^4^ and/or possible neurological damage from viruses^6^. Thus, the dose of clothianidin is critical for demonstrating an interaction between *V. destructor* and clothianidin on grooming intensity. It was also notable that the proportion of intense groomers was much more decreased by the stressors compared to the proportion of bees that groomed in any manner, possibly due to a greater effect on neural processes required for intense grooming, such as neurotransmitters like acetylcholine. Thus, future work is warranted to shed light on the neural processes involved in grooming and on differences in neurological activity between light and intense groomers. This appears to be first report of possible interactions between biotic and abiotic factors affecting grooming intensity in insects. Based on these results, it would be expected that colonies of honey bees exposed to *V. destructor* would be more susceptible to negative effects of clothianidin, possibly resulting in less resistance to *V. destructor* and thus larger populations of the parasite in the colonies, due to the impaired ability of the bees to groom themselves intensively.

Gene expression analysis using RNAseq with insect brains can be a powerful way to reveal impacts on biological pathways in the brain. Differential gene expression has been linked to pathogens, such as Black queen cell virus and *Nosema ceranae* in honey bees and nucleopolyhedrovirus in silkworms^41,42^ or behaviors, such as hygienic behavior in honey bees^43^.

One surprising result in this study from the RNAseq analysis was that the total number of DEGs significantly up-regulated uniquely by clothianidin was more than eleven times higher than with the combined stressors. Thus, the addition of *V. destructor* parasitism appeared to reduce the impacts of clothianidin on gene expression in the brain. One might have expected the reverse, where even more significantly up-regulated DEGs would be detected with the combined stressors due to effects of *V. destructor* parasitism making the bee more sensitive to clothianidin damage. The results could be due to *V. destructor* or viruses causing such strong damage to brain tissue that it limits the effects of clothianidin. This may also explain why the number of up-regulated DEGs was only slightly lower with *V. destructor* parasitism than by the combined stressors. This implies that the effects of combining stressors is unpredictable, at least for affecting gene expression in honey bee brains.

KEGG analysis provided some insights into the effects of clothianidin, *V. destructor* and clothianidin plus *V. destructor* on the brains of the treated bees. There were a number of DEGs that had KEGG terms that were unique to each stressor. KEGG terms solely observed with clothianidin exposure included Parkinson’s disease pathway, which is associated with degeneration of the central nervous system^44^, and sphingolipid metabolism, which is involved in signalling for diseases like the neurodegenerative Alzheimer’s disease^45^. Those terms indicate that sublethal effects of clothianidin on acetylcholine receptors may also be causing degeneration of the brain. KEGG terms solely found with *V. destructor* parasitism, included viral myocarditis that is involved in heart muscle inflammation commonly caused by viral infections^46^ and neuroactive ligand-receptor, which has been associated with the early stage symptoms of Parkinson’s disease in the *Drosophila* melanogaster disease model^47^. While *D. melanogaster* does not show the symptoms of Parkinson’s disease like humans, Whitworth^48^ noted that it is a good model as there is extensive conservation of neuronal function and development between *D. melanogaster* and vertebrates when examined at a cellular level. The association of those biological pathways with *V. destructor* parasitism indicates that certain impacts of the parasite or viruses transmitted by the parasite are detrimental to neural function. KEGG terms unique to the combined stressors included insulin signaling, whose disruption in brain tissue has been associated with neurodegenerative disorders, like Alzheimer’s disease in rats^49^. Insulin signaling has also been linked to cast differentiation^50^, longevity^51^ and division of labour^52^ in honey bees. Another KEGG term unique to the combined stressors was the NOD-like receptor signaling pathway, which regulates innate immune receptors whose disfunction is also associated with neurodegenerative disorders, like Alzheimer’s and Parkinson’s disease^53^. The association of those biological terms with the combined stressors suggest that the combined stressor treatment was resulting in DEGs related to neurodegeneration that were not detected with each stressor alone.

The fact that the three stressors shared common KEGG terms indicates some common impacts of clothianidin and *V. destructor* alone or in combination. KEGG terms shared between clothianidin and *V. destructor* included Alzheimer’s disease, which is a characterized by pathological alterations in neuronal receptors^54^, dopaminergic and glutamatergic synapse, which control functions like locomotor activity, learning and memory^55^, and peroxisome which is important in detoxification of free radicals that could damage neurological functions in brains^56^. KEGG terms found both with clothianidin and the combined stressors included cellular senescence related to an irreversible cellular arrest associated with brain pathology^57^. Among the KEGG terms associated with both *V. destructor* and the combined stressors were hippo signalling pathway-fly related to cell proliferation and whose dysregulation is linked to pathologies like cancer^58^. The KEGG term longevity regulating pathway-multiple species was found with all the three treatments. That biological pathway promotes cellular fitness through autophagy and stress defence^59^. All of the above indicates that a parasite and sub-lethal doses of a neonicotinoid have some shared as well as unique negative impacts on the honey bee brain, like cell damage, proliferation and senescence, that are consistent with aspects of neurodegeneration.

In conclusion, this study showed that a sublethal, chronic exposure to clothianidin, similar to that expected under field conditions, can more negatively impact the self-grooming behavior of honey bees when combined with *V. destructor* parasitism than applied alone. Additionally, RNAseq analysis of the brains of treated bees revealed different impacts on gene expression by each stressor, which was also observed when the stressors were combined based on the number of DEGs. Surprisingly, the interaction between the stressors decreased rather than increased the number of up and down-regulated DEGs found with clothianidin. Although a variety of biological pathways were associated with the DEGs, it was notable that many terms were associated with neurodegeneration and cell damage implying that each stressor alone or in combination may negatively affect neural activity, which could help explain their impact on grooming behavior potentially reducing the bees’ survival.

## Methods

### Sources of honey bees and varroa mites

Honey bees were obtained from colonies of the Buckfast strain at the University of Guelph’s Honey Bee Research Centre in Ontario, Canada. The queens of the colonies for the brood and workers in this study were mated under controlled conditions in isolation at Thorah Island, Simcoe, ON, to guarantee the purity of the Buckfast strain and uniformity of its genotype. The colonies that were used as source of bees were not previously exposed to treatments, such as pesticides. The newly emerged bees used for the four different biological repetitions did not come from the same colony, but the bees shared the same genotype. Female *Varroa* mites were collected from highly infested colonies as per Arechavaleta and Guzman-Novoa^11^ and placed in a Petri dish for immediate use in experiments.

### Working clothianidin dilutions

Clothianidin (Sigma Aldrich®, Oakville, ON, Canada) was applied at 9 × 10^−4^ ng/µl, 4.2 × 10^−3^ ng/µl and 1 × 10^−2^. These were based on a honey bee consuming approx. 25.5–39 mg nectar per day^60^ and 0.0012–0.0086 ng/mg clothianidin being in the nectar of canola grown from clothianidin treated seeds^61–63^. Thus, a honey bee could consume 0.03–0.34 ng clothianidin per day ($\bar{x}$ = 0.137, SE = 0.028).

### Exposure to clothianidin and/or *V. destructor*

Newly emerged bees (<24 h) were obtained from frames after incubation in screened emerging cages (50.3 × 7.3 × 25.2 cm) at 35 °C and 60% RH overnight. Forty newly-emerged bees were randomly assigned to each of the eight treatments. The treatments were 0 ng/µl (control), 9 × 10^−4^ ng/µl, 4.2 × 10^−3^ ng/µl, 1 × 10^−2^ ng/µl, 0 ng/µl + *V. destructor*, 9 × 10^−4^ ng/µl + *V. destructor*, 4.2 × 10^−3^ ng/µl + *V. destructor*, and 1 × 10^−2^ ng/µl + *V. destructor*. For the four treatments with *V. destructor*, one female mite was taken from a Petri dish using a fine paintbrush, placed on the abdomen or thorax of a bee and observed to verify that the mite was attached to the bee’s body. The bees were incubated in a sterilized hoarding cage (12.7 × 8.5 × 14.5 cm) at 35 °C and 60% RH for seven consecutive days and were fed 50% sugar syrup *ad libitum* containing the desired dose of clothianidin, using a 20 ml gravity feeder. The sugar syrup contained in the feeders was weighed on the 3^rd^ and 7^th^ day of treatment to determine the amount of syrup that had been consumed by the 40 bees in each hoarding cage, which was within the amounts reported in the literature. The mortality of the bees was also recorded at the end of the 7th day of treatment, but it was negligible, as it was less than 2% in all the treatment groups. The experiment was repeated four times. The treatments that corresponded to the same biological repetition were done at the same time.

### Grooming behavior assays

Seven days after treatment, 1,056 bees from the eight treatments were assessed for self-grooming behavior as per Aumeier^64^ with some modifications^65^. Briefly, a treated bee was placed in a Petri dish (100 mm × 15 mm, FisherScientific® Mississauga, ON, Canada) that was lined with Whatman™ white filter paper (Fisher Scientific®) covered with perforated plastic foil. After being introduced into the Petri dish, the bee remained for 1 min to become accustomed to the testing arena. Then, approximately 20 mg of wheat flour were put on the dorsal surface of the bee’s thorax using a fine brush, and for 3 min, self-grooming instances exhibited were recorded and classified as per Guzman-Novoa *et al*.^10^ A study conducted by Espinosa-Montaño^65^ showed that using 20 mg of flour was as reliable as using a varroa mite as an irritant for the grooming behavior assay. Class “light grooming” occurred if slow swipes were observed and the bee used one leg or two legs at most, class “intense grooming” occurred if the bee performed vigorous wiping and shaking using more than two legs, and class “no grooming” occurred if the bee did not show any kind of grooming activity. After the assessments, all the bees used in the trials were immediately frozen at −70 °C for RNA analysis.

### RNA extraction and RNAseq analysis

Total RNA was extracted from 15 to 25 brains obtained from randomly selected bees per treatment for three replications, using TRIzol® reagent following the manufacturer’s instructions with modifications as per Boutin *et al*.^43^ 15 µl of the RNA from each of the three biological replicates per treatment were pooled to obtain the equivalent of RNA from 45 brains, which was used for RNAseq analysis. RNA samples were sent to McGill University (Génome Québec Innovation Centre, Montreal, QC, Canada) to perform a high throughput sequencing analysis, using a HiSeq. 2500 v4 (Illumina, San Diego, CA, USA).

### DWV quantification

15 µl of the RNA from three biological replicates per treatment was also used for DWV quantification. To calculate the number of DWV genome copies per sample, primers (5′-3′ F: GGGTAACGTGCGACGTTTTA; R: GACGTAAAGGCGGTAGTTGC) specific for the DWV helicase were used^33^. PCR conditions were one cycle at 48 °C for 15 min, one at 95 °C for 10 min, 40 at 95 °C for 15 s and 60 °C for 60 s, followed by one cycle at 68 °C for 7 min. The reaction volume was 25 µl containing 2 µl template, 3 µl 200 nM primers,12.5 µl Maxima SYBR Green/ROX qRT-PCR Master Mix (2×) and 9.5 µl nuclease free H_2_O per sample. As a negative control, nuclease free H_2_O was included instead of cDNA, and a positive control from previously identified DWV positive bee samples by qRT-PCR were included in each qRT-PCR run. Calibration curves to convert Ct values to DWV genome copies were done using 300 bp gBlocks® (Integrated DNA Technologies, IA, USA) that included the sequence of the forward primer, amplicon and reverse primer. The lyophilized gBlock® was diluted with 20 µl of ds H_2_0 to obtain an initial concentration of 10 ng/µl that was used to make serial dilutions from 10^9^ to 10^1^ copies. Using a plot of Ct values versus DWV copy number (log_10_), a linear equation was used to calculate the DWV genome copy numbers for each of the samples of interest^66^.

### Statistical analyses

To compare the proportions of the grooming classes, contingency tables using Chi^2^ tests of independence with α of 0.05 were used, and adjusted residuals were calculated for *post hoc* analysis. DWV quantities were tested with the Shapiro Wilk test and transformed to a base 10 logarithm (due to lack of normality) before being subjected to two-way ANOVAs and Tukey HSD tests with α of 0.05. The above statistical analyses were performed using R, version 3.4.3© (The R Foundation for Statistical Computing, 2017).

RNAseq was performed at Génome Québec Innovation (Montreal, QC, Canada). Library preparation was done using the NEB kit Illumina (San Diego, CA, USA) for poly(A)+ enriched RNA prepared with a KAPA kit (Roche, Mississauga, ON, Canada) according to the manufacturer’s instructions. Sequencing was performed as 125 bp, paired-end reads using a HiSeq2500 v4 (Illumina, San Diego, CA, USA).

Bioinformatic analysis was performed at the Canadian Centre for Computational Genomics (C3G) (Montreal, QC, Canada). Sequence trimming was done with Trimmomatic software^67^. Read sets were aligned to a reference genome of the honey bee, *Apis mellifera* (ftp://ftp.ncbi.nlm.nih.gov/genomes/Apis_mellifera) (ver Amel_4.5) using STAR^68^. Aligned RNAseq reads were assembled into transcripts, and fragments per kilobase of exon per million fragments mapped (FPKM) was determined with Cufflinks^69^. Differential gene analysis (DGA) to identify differentially expressed genes (DEGs) was done using the DESeq R Bioconductor package^70^ and edgeR Bioconductor package^71^. Transcript expression levels and test for significant differences (P < 0.05) was calculated with Cuffdiff ^69^ using the FPKM values.

The pairwise comparisons of DEGs was for bees exposed to 0 ng of clothianidin vs 1 × 10^−2^ ng/µl of clothianidin (0 vs 1 × 10^−2^), 0 ng of clothianidin vs *V. destructor* (0 vs Vd) and 0 ng of clothianidin vs 1 × 10^−2^ ng/µl of clothianidin plus *V. destructor* (0 vs 1 × 10^−2^ + Vd). Biological pathways of the DEGs were determined by the KASS-KEGG automatic annotation server^72^ with the Kyoto Encyclopaedia of Genes and Genomes (KEGG)^73^ by inputting the nucleotide sequences of the DEGs. Venn diagrams were created with the DEGs from the pairwise comparison using the Bioinformatics and Evolutionary Genomics website (http://bioinformatics.psb.ugent.be/beg/tools/venn-diagrams)^74–77^.

## Supplementary information

Supplementary Tables

## Data Availability

The datasets generated from grooming behavior during the current study are available from the corresponding author on reasonable request. All other data generated or analysed during this study are included in this published article (and its Supplementary Information files).

## References

[1] Webb B (2012). Cognition in insects. Philos. Trans. R. Soc. Lond. B. Biol. Sci..

[2] Le Conte Y, Ellis M, Ritter W (2010). *Varroa* mites and honey bee health: can varroa explain part of the colony losses?. Apidologie.

[3] Vidal-Naquet, N. & Lewbart, G. Parasitic diseases in *Honeybee veterinary medicine*: *Apis mellifera* L. (ed. Vidal-Naquet, N.) 109–144 (5M Publishing, 2015).

[4] Rosenkranz P, Aumeier P, Ziegelmann B (2010). Biology and control of *Varroa destructor*. J. Invertebr. Patho..

[5] Koleoglu G, Goodwin PH, Reyes-Quintana M, Hamiduzzaman MM, Guzman-Novoa E (2017). Effect of *Varroa destructor*, wounding and *Varroa* homogenate on gene expression in brood and adult honey bees. PLoS One.

[6] Iqbal J, Mueller U (2007). Virus infection causes specific learning deficits in honeybee foragers. Proc. R. Soc.Lond. B. Biol. Sci..

[7] Anguiano-Baez, R., Guzman-Novoa, E., Espinosa-Montaño, L. G. & Correa-Benítez, A. *Varroa destructor* (Mesostigmata: Varroidae) parasitism and climate differentially influence the prevalence, levels, and overt infections of deformed wing virus in honey bees (Hymenoptera: Apidae). *J. Insect Sci*. **16**, 10.1093/jisesa/iew029 (2016). 10.1093/jisesa/iew029 PMC4887826 27252482

[8] Boecking O, Spivak M (1999). Behavioral defenses of honey bees against *Varroa jacobsoni* Oud. Apidologie.

[9] Peng YS, Fang Y, Xu S, Ge L (1987). The resistance mechanism of the Asian honey bee, *Apis cerana* Fabr., to an ectoparasitic mite, *Varroa jacobsoni* Oudemans. J. Invertebr. Patho..

[10] Guzman-Novoa E, Emsen B, Unger P, Espinosa-Montaño LG, Petukhova T (2012). Genotypic variability and relationships between mite infestation levels, mite damage, grooming intensity, and removal of *Varroa destructor* mites in selected strains of worker honey bees (*Apis mellifera* L.). J. Invertebr. Patho..

[11] Arechavaleta-Velasco M, Guzman-Novoa E (2001). Relative effect of four characteristics that restrain the population growth of the mite *Varroa destructor* in honey bee (*Apis mellifera*) colonies. Apidologie.

[12] Currie RW, Tahmasbi GH (2008). The ability of high-and low-grooming lines of honey bees to remove the parasitic mite *Varroa destructor* is affected by environmental conditions. Can. J. Zool..

[13] Arechavaleta-Velasco ME, Alcala-Escamilla K, Robles-Rios C, Tsuruda JM, Hunt GJ (2012). Fine-scale linkage mapping reveals a small set of candidate genes influencing honey bee grooming behavior in response to *Varroa* mites. PLoS One.

[14] Hamiduzzaman MM (2017). Differential gene expression associated with honey bee grooming behavior in response to *Varroa* mites. Behav. Genet..

[15] Simon, J. Y. *The toxicology and biochemistry of insecticides* 1–380 (CRC press, 2011).

[16] Knowles CO, Casida JE (1966). Mode of action of organophosphate anthelmintics. Cholinesterase inhibition in *Ascaris lumbricoides*. J. Agric. Food Chem..

[17] Vijverberg HP, vanden Bercken J (1990). Neurotoxicological effects and the mode of action of pyrethroid insecticides. Crit. Rev. Toxicol..

[18] Nauen R, Bretschneider T (2002). New modes of action of insecticides. Pestic. Outlook.

[19] Haynes KF (1988). Sublethal effects of neurotoxic insecticides on insect behavior. Annu. Rev. Entomol..

[20] Aliouane Y (2009). Subchronic exposure of honeybees to sublethal doses of pesticides: effects on behavior. Environ. Toxicol. Chem..

[21] Decourtye A, Lacassie E, Pham-Delègue MH (2003). Learning performances of honeybees (*Apis mellifera* L.) are differentially affected by imidacloprid according to the season. Pest Manag. Sci..

[22] Williamson SM, Willis SJ, Wright GA (2014). Exposure to neonicotinoids influences the motor function of adult worker honeybees. Ecotoxicology.

[23] Stanley DA, Smith KE, Raine NE (2015). Bumblebee learning and memory is impaired by chronic exposure to a neonicotinoid pesticide. Sci. Rep..

[24] Pisa LW (2015). Effects of neonicotinoids and fipronil on non-target invertebrates. Environ. Sci. Pollut. Res..

[25] Hopwood, J. A. *et al*. *How neonicotinoids can kill bees: the science behind the role these insecticides play in harming bees 2*^*nd*^*Ed*. 1–76 (The Xerces Society for Invertebrate Conservation, 2016).

[26] Fussnecker BL, Smith BH, Mustard JA (2006). Octopamine and tyramine influence the behavioral profile of locomotor activity in the honey bee (*Apis mellifera*). J. Insect Physiol..

[27] Mustard JA, Pham PM, Smith BH (2010). Modulation of motor behavior by dopamine and the D1-like dopamine receptor AmDOP2 in the honey bee. J. Insect Physiol..

[28] Mattson MP (2008). Hormesis defined. Ageing Res. Rev..

[29] Yamamoto, I. Nicotine to Nicotinoids: 1962 to 1997 in *Nicotinoid insecticides and the nicotinic acetylcholine receptor* (ed Yamamoto, I. & Casida, J. E.) 3–27 (Springer, 1999).

[30] Bowen-Walker PL, Martin SJ, Gunn A (1999). The transmission of deformed wing virus between honeybees (*Apis mellifera* L.) by the ectoparasitic mite *Varroa jacobsoni* Oud. J. Invertebr. Pathol..

[31] Hamiduzzaman MM (2015). Differential responses of Africanized and European honey bees (*Apis mellifera*) to viral replication following mechanical transmission or *Varroa destructor* parasitism. J. Invertebr. Pathol..

[32] Emsen B, Guzman-Novoa E, Kelly PG (2014). Honey production of honey bee (Hymenoptera: Apidae) colonies with high and low *Varroa destructor* (Acari: Varroidae) infestation rates in eastern Canada. Can. Entomol..

[33] Di Prisco G (2013). Neonicotinoid clothianidin adversely affects insect immunity and promotes replication of a viral pathogen in honey bees. Proc. Natl. Acad. Sci. USA.

[34] Tak PP, Firestein GS (2001). NF-κB: a key role in inflammatory diseases. J. Clin. Invest..

[35] Brandt A, Gorenflo A, Siede R, Meixner M, Büchler R (2016). The neonicotinoids thiacloprid, imidacloprid, and clothianidin affect the immunocompetence of honey bees (*Apis mellifera* L.). J. Insect Physiol..

[36] Lamiable O (2016). Analysis of the contribution of hemocytes and autophagy to *Drosophila* antiviral immunity. J. Virol..

[37] Shah KS, Evans EC, Pizzorno MC (2009). Localization of deformed wing virus (DWV) in the brains of the honeybee, *Apis mellifera* Linnaeus. Virol. J..

[38] Platt KB (1997). Impact of dengue virus infection on feeding behavior of *Aedes aegypti*. Am. J. Trop. Med. Hyg..

[39] de Roode JC, Lefèvre T (2012). Behavioral immunity in insects. Insects..

[40] Smith DS, Treherne JE (1963). Functional aspects of the organization of the insect nervous system. Adv. In Insect. Phys..

[41] Doublet V (2016). Brain transcriptomes of honey bees (*Apis mellifera*) experimentally infected by two pathogens: black queen cell virus and *Nosema ceranae*. Genomics Data..

[42] Wang G (2015). Transcriptome analysis of the brain of the silkworm *Bombyx mori* infected with *Bombyx mori* nucleopolyhedrovirus: A new insight into the molecular mechanism of enhanced locomotor activity induced by viral infection. J. Invertebr. Pathol..

[43] Boutin S, Alburaki M, Mercier PL, Giovenazzo P, Derome N (2015). Differential gene expression between hygienic and non-hygienic honeybee (*Apis mellifera* L.) hives. BMC Genomics.

[44] McGeer PL, McGeer EG (2004). Inflammation and the degenerative diseases of aging. Ann. N. Y. Acad. Sci..

[45] Pralhada Rao, R. *et al*. Sphingolipid metabolic pathway: an overview of major roles played in human diseases. *J*. *Lipids*. **178910**, 10.1155/2013/178910 (2013). 10.1155/2013/178910 PMC3747619 23984075

[46] Esfandiarei M, McManus BM (2008). Molecular biology and pathogenesis of viral myocarditis. Annu. Rev. Pathmechdis. Mech. Dis..

[47] Kong Y (2015). L. High throughput sequencing identifies MicroRNAs mediating α-synuclein toxicity by targeting neuroactive-ligand receptor interaction pathway in early stage of *Drosophila* Parkinson’s disease model. PLoS One.

[48] Whitworth, A. J. *Drosophila* models of Parkinson’s disease in *Advances in genetics* (ed. Dhevendra Kumar) 73, 1–50 (Academic Press, 2011). 10.1016/B978-0-12-380860-8.00001-X 21310293

[49] Craft S, Watson GS (2004). Insulin and neurodegenerative disease: shared and specific mechanisms. Lancet Neurol..

[50] de Azevedo SV, Hartfelder K (2008). The insulin signaling pathway in honey bee (*Apis mellifera*) caste development—differential expression of insulin-like peptides and insulin receptors in queen and worker larvae. J. Insect Physiol..

[51] Corona M (2007). Vitellogenin, juvenile hormone, insulin signaling, and queen honey bee longevity. Proc. Natl. Acad. Sci..

[52] Ament SA, Corona M, Pollock HS, Robinson GE (2008). Insulin signaling is involved in the regulation of worker division of labor in honey bee colonies. Proc. Natl. Acad. Sci.

[53] Kong X, Yuan Z, Cheng J (2017). The function of NOD‐like receptors in central nervous system diseases. J. Neurosci. Res..

[54] Xu Y (2012). Neurotransmitter receptors and cognitive dysfunction in Alzheimer’s disease and Parkinson’s disease. Prog. Neurobiol..

[55] Beaulieu JM, Gainetdinov RR (2011). The physiology, signaling, and pharmacology of dopamine receptors. Pharmacol. Rev..

[56] Siesjö BK, Agardh CD, Bengtsson F (1989). Free radicals and brain damage. Cerebrovasc. Brain. Metab. Rev..

[57] Coyle JT, Puttfarcken P (1993). Oxidative stress, glutamate, and neurodegenerative disorders. Science.

[58] Pan D (2010). The hippo signaling pathway in development and cancer. Dev. Cell.

[59] Kenyon C (2005). The plasticity of aging: insights from long-lived mutants. Cell.

[60] Winston, M. L. Development and nutrition in *The Biology of the honey bee* (ed. Winston, M. L.) 46–71 (University Press, 1991).

[61] Cutler GC, Scott-Dupree CD (2007). Exposure to clothianidin seed-treated canola has no long-term impact on honey bees. J. Econ. Entomol..

[62] European Food Safety Authority (EFSA) (2013). Conclusion on the peer review of the pesticide risk assessment for bees for the active substance clothianidin. EFSA J..

[63] Pilling E, Campbell P, Coulson M, Ruddle N, Tornier I (2013). A four-year field program investigating long-term effects of repeated exposure of honey bee colonies to flowering crops treated with thiamethoxam. PLoS One.

[64] Aumeier P (2001). Bioassay for grooming effectiveness towards *Varroa destructor* mites in Africanized and Carniolan honey bees. Apidologie.

[65] Espinosa-Montaño, L. G. Heredabilidades y correlaciones fenotípicas para algunas características que influyen en la resistencia de las abejas (*Apis mellifera*) al crecimiento poblacional del ácaro *Varroa destructor* en México. (Doctoral Thesis). Universidad Nacional Autónoma de México, Mexico City (2006).

[66] Bustin SA (2009). The MIQE guidelines: minimum information for publication of quantitative real-time PCR experiments. Clin. Chem..

[67] Bolger AM, Lohse M, Usadel B (2014). Trimmomatic: a flexible trimmer for Illumina sequence data. Bioinformatics.

[68] Dobin A (2013). STAR: ultrafast universal RNA-seq aligner. Bioinformatics.

[69] Roberts A, Pimentel H, Trapnell C, Pachter L (2011). Identification of novel transcripts in annotated genomes using RNA-Seq. Bioinformatics.

[70] Anders S, Huber W (2010). Differential expression analysis for sequence count data. Genome Biol..

[71] Robinson MD, McCarthy DJ, Smyth G (2010). K. edgeR: a Bioconductor package for differential expression analysis of digital gene expression data. Bioinformatics.

[72] Moriya Y, Itoh M, Okuda S, Yoshizawa AC, Kanehisa M (2007). KAAS: an automatic genome annotation and pathway reconstruction server. Nucleic Acids Res..

[73] Kanehisa M, Goto S (2000). KEGG: Kyoto Encyclopedia of Genes and Genomes. Nucleic Acids Res..

[74] Elsik CG (2014). Finding the missing honey bee genes: lessons learned from a genome upgrade. BMC Genomics.

[75] Elsik CG (2016). Hymenoptera Genome Database: integrating genome annotations in HymenopteraMine. Nucleic Acids Res..

[76] Benson DA (2012). GenBank. Nucleic Acids Res..

[77] Reimand J, Arak T, Vilo J (2011). g: Profiler a web server for functional interpretation of gene lists. Nucleic Acids Res..

