# Supplementary material for: Effects of sublethal doses of clothianidin and/or V. destructor on honey bee (Apis mellifera) self-grooming behavior and associated gene expression
Source: Sci Rep. 2019 Mar 26;9:5196. doi: 10.1038/s41598-019-41365-0 (PMC6435647; doi:10.1038/s41598-019-41365-0)
Supplement: Supplementary file 1 — Supplementary Tables [file 41598_2019_41365_MOESM1_ESM.docx]

Effects of sublethal doses of clothianidin and/or *V. destructor* on honey bee (*Apis mellifera*) self-grooming behavior and associated gene expression

Nuria Morfin, Paul H. Goodwin, Greg J. Hunt & Ernesto Guzman-Novoa

**Table S1**. Significantly up-regulated DEGs in bees exposed to 1X10^-2^ ng of clothianidin per µl compared to the bees exposed to 0 ng of clothianidin (0vs1X10^-2^) (Pearson pairwise comparison, p<0.05).

| **Gene ID^a^** | **Gene description^b^** | **LogFC^c^** |
| --- | --- | --- |
| GB48373 | transmembrane protein C9orf91 homolog | 7.59 |
| GB51202 | zinc finger MYND domain-containing protein 10 homolog | 6.66 |
| GB54954 | DNA primase large subunit-like | 4.11 |
| GB43169 | phosphatidylinositol N-acetylglucosaminyltransferase subunit C | 3.83 |
| GB41338 | venom acid phosphatase | 3.54 |
| GB51001 | dnaJ homolog subfamily C member 4-like | 3.35 |
| GB46595 | uncharacterized | 3.28 |
| GB51061 | exostosin-1 | 3.20 |
| GB54955 | ER membrane protein complex subunit 8/9 homolog | 3.08 |
| GB42704 | takeout-like | 3.03 |
| GB52244 | telomerase reverse transcriptase | 3.00 |
| GB40546 | translation initiation factor IF-3-like | 2.94 |
| GB48922 | uncharacterized membrane protein | 2.86 |
| GB55436 | uncharacterized | 2.85 |
| GB45312 | uncharacterized | 2.73 |
| GB41099 | uncharacterized | 2.72 |
| GB55110 | 7-methylguanosine phosphate-specific 5'-nucleotidase | 2.71 |
| GB41078 | dolichol-phosphate mannosyltransferase subunit 3 | 2.67 |
| GB45861 | uncharacterized | 2.64 |
| GB47618 | defensin 2 | 2.64 |
| GB51602 | 39S ribosomal protein L34, mitochondrial | 2.63 |
| GB52560 | penguin | 2.62 |
| GB42050 | uncharacterized | 2.56 |
| GB45422 | transmembrane protein 231 | 2.56 |
| GB48084 | ethanolamine kinase 1 | 2.53 |
| GB47487 | oligoribonuclease | 2.50 |
| GB52235 | uncharacterized | 2.44 |
| GB52470 | ribonuclease H2 subunit C | 2.39 |
| GB51338 | cilia- and flagella-associated protein 69-like | 2.38 |
| GB41973 | thioredoxin domain-containing protein 9 | 2.35 |
| GB52577 | Werner exonuclease | 2.29 |
| GB54400 | elongation of very long chain fatty acids protein AAEL008004-like | 2.28 |
| GB45366 | uncharacterized | 2.28 |
| GB52888 | uncharacterized | 2.22 |
| GB46706 | uncharacterized | 2.20 |
| GB53649 | dual specificity protein phosphatase 19-like | 2.20 |
| GB53200 | dnaJ homolog subfamily C member 18-like | 2.18 |
| GB44264 | transmembrane protein 70 homolog | 2.17 |
| GB40810 | transmembrane protein 70 homolog | 2.16 |
| GB42615 | uncharacterized | 2.16 |
| GB50550 | uncharacterized | 2.15 |
| GB47347 | uncharacterized | 2.14 |
| GB48581 | peptidyl-prolyl cis-trans isomerase-like 6 | 2.13 |
| GB48379 | uncharacterized | 2.13 |
| GB54212 | uncharacterized | 2.10 |
| GB42380 | E3 ubiquitin-protein ligase RNF126 | 2.10 |
| GB55810 | uncharacterized | 2.09 |
| GB41925 | uncharacterized | 2.09 |
| GB43639 | uncharacterized | 2.09 |
| GB44068 | protein bcn92 | 2.09 |
| GB52685 | cattle cerebrum and skeletal muscle-protein 1 | 2.07 |
| GB45210 | translocon-associated protein subunit gamma-like | 2.04 |
| GB46881 | COMM domain-containing protein 5-like | 2.04 |
| GB42964 | beta-1,3-glucosyltransferase | 2.00 |
| GB41809 | NADH dehydrogenase [ubiquinone] 1 alpha subcomplex assembly factor 3 | 1.99 |
| GB51990 | uncharacterized | 1.98 |
| GB48954 | uncharacterized membrane protein | 1.98 |
| GB50413 | transforming growth factor beta regulator 4 | 1.95 |
| GB41173 | uncharacterized | 1.95 |
| GB55157 | prefoldin subunit 4 | 1.94 |
| GB50033 | COMM domain-containing protein 2 | 1.94 |
| GB49371 | NADH-cytochrome b5 reductase-like | 1.92 |
| GB54293 | uncharacterized | 1.92 |
| GB45122 | mitochondrial assembly of ribosomal large subunit protein 1 | 1.92 |
| GB44571 | PQ-loop repeat-containing protein 1 | 1.89 |
| GB52011 | myb-binding protein 1A-like | 1.89 |
| GB52805 | phosphatidylinositol N-acetylglucosaminyltransferase subunit Q | 1.89 |
| GB55149 | uncharacterized membrane protein | 1.82 |
| GB46182 | uncharacterized | 1.81 |
| GB49414 | post-GPI attachment to proteins factor 3 | 1.81 |
| GB47595 | sine oculis-binding protein homolog | 1.81 |
| GB48835 | EKC/KEOPS complex subunit TPRKB-like | 1.80 |
| GB55939 | beta-1,3-galactosyltransferase 5 | 1.80 |
| GB42754 | uncharacterized | 1.78 |
| GB41026 | uncharacterized | 1.77 |
| GB50343 | ribonuclease P protein subunit p29 | 1.75 |
| GB45697 | uncharacterized | 1.75 |
| GB51601 | tRNA (guanine-N(7)-)-methyltransferase non-catalytic subunit WDR4 | 1.75 |
| GB54655 | GDP-fucose transporter 1 | 1.74 |
| GB40903 | uncharacterized membrane protein | 1.74 |
| GB43783 | uncharacterized | 1.73 |
| GB54469 | ribosome-recycling factor | 1.73 |
| GB46563 | protein dopey homolog PFC0245c-like | 1.69 |
| GB54572 | ribonuclease P protein subunit p30-like | 1.69 |
| GB47082 | DALR anticodon-binding domain-containing protein 3-like | 1.67 |
| GB43900 | alpha-ketoglutarate-dependent dioxygenase alkB homolog 7 | 1.67 |
| GB42015 | tubulin polyglutamylase TTLL7-like | 1.66 |
| GB47207 | thioredoxin | 1.66 |
| GB45085 | transmembrane protein 177 | 1.65 |
| GB47337 | uncharacterized | 1.62 |
| GB50346 | translation initiation factor eIF-2B subunit epsilon | 1.61 |
| GB47177 | ATP-dependent RNA helicase TDRD12 | 1.60 |
| GB55191 | uncharacterized | 1.60 |
| GB41153 | peroxisomal membrane protein PEX16 | 1.58 |
| GB51272 | MATH and LRR domain-containing protein PFE0570w-like | 1.55 |
| GB44504 | uncharacterized | 1.55 |
| GB42653 | glycogenin- | 1.54 |
| GB51637 | major facilitator superfamily domain-containing protein 12-like | 1.53 |
| GB51223 | hymenoptaecin | 1.51 |
| GB49234 | uncharacterized | 1.51 |
| GB43822 | KRTCAP2 homolog | 1.49 |
| GB52190 | tRNA pseudouridine synthase A | 1.47 |
| GB52245 | speckle targeted PIP5K1A-regulated poly(A) polymerase-like | 1.47 |
| GB51606 | timeless interacting homolog | 1.47 |
| GB41490 | uncharacterized | 1.47 |
| GB43784 | uncharacterized | 1.47 |
| GB43946 | protein PF14_0175-like | 1.45 |
| GB48484 | 28S ribosomal protein S33 | 1.45 |
| GB54420 | zinc finger protein DZIP1 | 1.45 |
| GB53193 | PRKR-interacting protein 1 homolog | 1.44 |
| GB49385 | uncharacterized | 1.43 |
| GB55111 | TP53-regulated inhibitor of apoptosis 1-like | 1.42 |
| GB54050 | chitobiosyldiphosphodolichol beta-mannosyltransferase-like | 1.42 |
| GB42631 | uncharacterized | 1.42 |
| GB43510 | pancreatic triacylglycerol lipase-like | 1.41 |
| GB55298 | cytochrome b-c1 complex subunit 9 | 1.39 |
| GB48578 | lethal(2)neighbour of Tid | 1.39 |
| GB50323 | phosphatidylinositol glycan anchor biosynthesis class U | 1.39 |
| GB41118 | carbonic anhydrase 2-like | 1.37 |
| GB42419 | RNA polymerase II subunit B1 CTD phosphatase Rpap2 | 1.37 |
| GB49524 | GPI mannosyltransferase 3 | 1.37 |
| GB45355 | uncharacterized | 1.36 |
| GB54610 | thiamine transporter 2-like | 1.35 |
| GB54091 | testis-expressed sequence 10 protein homolog | 1.34 |
| GB51306 | apidaecins type 73 | 1.33 |
| GB46534 | uncharacterized | 1.31 |
| GB45653 | membrane magnesium transporter 1 | 1.31 |
| GB51367 | tRNA (guanine(37)-N1)-methyltransferase | 1.30 |
| GB47315 | mitochondrial thiamine pyrophosphate carrier-like | 1.30 |
| GB53663 | GTPase Era | 1.28 |
| GB54960 | oligoribonuclease | 1.28 |
| GB43815 | 39S ribosomal protein L23 | 1.28 |
| GB50641 | enkurin domain-containing protein 1 | 1.28 |
| GB54602 | gamma-tubulin complex component 5 | 1.27 |
| GB52638 | ninjurin-1-like | 1.26 |
| GB42376 | bud22-like | 1.25 |
| GB51535 | MATH and LRR domain-containing protein PFE0570w-like | 1.24 |
| GB51627 | dehydrodolichyl diphosphate syntase complex subunit DHDDS (LOC551358), transcript variant X3 | 1.24 |
| GB42919 | Fanconi anemia group D2 protein | 1.23 |
| GB43193 | origin recognition complex subunit 1-like | 1.23 |
| GB50423 | immune responsive protein 30 | 1.23 |
| GB40890 | proteasome assembly chaperone 2-like | 1.23 |
| GB40884 | protein saal1 | 1.22 |
| GB53028 | laccase-1-like | 1.22 |
| GB55156 | THUMP domain-containing protein 1 homolog | 1.21 |
| GB40562 | zinc finger protein 724 | 1.20 |
| GB54097 | malvolio | 1.18 |
| GB50005 | Kazal-type serine protease inhibitor | 1.18 |
| GB44514 | UDP-xylose and UDP-N-acetylglucosamine transporter | 1.17 |
| GB42621 | fibroin heavy chain | 1.15 |
| GB44641 | F-box/WD repeat-containing protein 9 | 1.14 |
| GB42083 | uncharacterized | 1.12 |
| GB42383 | uncharacterized | 1.12 |
| GB44901 | conserved oligomeric Golgi complex subunit 2 | 1.12 |
| GB43561 | uncharacterized | 1.12 |
| GB47546 | apidaecin precursor | 1.12 |
| GB51719 | ribonuclease Z | 1.11 |
| GB49956 | proline synthase co-transcribed bacterial homolog protein | 1.11 |
| GB55457 | C2 domain-containing protein 3 | 1.10 |
| GB48923 | microfibrillar-associated protein 1 | 1.09 |
| GB49582 | double-strand break repair protein MRE11 | 1.08 |
| GB41806 | calcyphosin-like | 1.08 |
| GB47538 | cytochrome b reductase 1-like | 1.08 |
| GB50213 | PTCD3 homolog | 1.05 |
| GB51132 | SYS1 homolog | 1.05 |
| GB50352 | glutathione synthetase | 1.04 |
| GB41977 | m7GpppX diphosphatase | 1.04 |
| GB41976 | zinc finger protein 567 | 1.04 |
| GB53876 | poly(U)-specific endoribonuclease homolog | 1.04 |
| GB44455 | uncharacterized | 1.00 |
| GB42650 | GPI mannosyltransferase 4 | 0.99 |
| GB52744 | poly(A) RNA polymerase | 0.99 |
| GB45125 | mRNA cap guanine-N7 methyltransferase | 0.99 |
| GB54611 | ovalbumin-related protein X | 0.98 |
| GB43134 | cleft lip and palate transmembrane protein 1-like | 0.97 |
| GB47571 | dihydroxyacetone phosphate acyltransferase | 0.96 |
| GB49086 | folylpolyglutamate synthase | 0.96 |
| GB45228 | chondroitin sulfate synthase 2 | 0.95 |
| GB41142 | dolichyl pyrophosphate Glc1Man9GlcNAc2 alpha-1,3-glucosyltransferase | 0.94 |
| GB41339 | venom acid phosphatase 1-like | 0.94 |
| GB52673 | gamma-tubulin complex component 6 | 0.93 |
| GB43418 | uncharacterized | 0.92 |
| GB50420 | TELO2-interacting protein 1 homolog | 0.92 |
| GB45808 | uncharacterized | 0.92 |
| GB47108 | myb-like protein D | 0.91 |
| GB43540 | pentatricopeptide repeat-containing protein 2 | 0.91 |
| GB44144 | phenylalanine--tRNA ligase | 0.91 |
| GB47459 | uncharacterized | 0.90 |
| GB40800 | signal peptidase complex subunit 3 | 0.88 |
| GB42081 | MATH and LRR domain-containing protein PFE0570w-like | 0.87 |
| GB48408 | catecholamines up | 0.87 |
| GB50253 | peptide deformylase | 0.87 |
| GB43901 | DNA replication ATP-dependent helicase/nuclease DNA2 | 0.86 |
| GB42958 | early endosome antigen 1-like | 0.86 |
| GB50039 | methionine--tRNA ligase | 0.86 |
| GB55979 | ribonuclease P protein subunit p40-like | 0.85 |
| GB55826 | condensin-2 complex subunit D3-like | 0.85 |
| GB41657 | uncharacterized | 0.85 |
| GB49642 | DNA-dependent protein kinase catalytic subunit-like | 0.84 |
| GB48820 | antitrypsin-like | 0.84 |
| GB53798 | esterase E4-like | 0.84 |
| GB40727 | CDP-diacylglycerol--inositol 3-phosphatidyltransferase | 0.83 |
| GB46267 | uncharacterized | 0.83 |
| GB40782 | ribosomal RNA processing protein 1 homolog | 0.81 |
| GB54108 | dual specificity protein phosphatase 3 | 0.80 |
| GB50883 | gem-associated protein 7-like | 0.80 |
| GB43485 | peroxisomal biogenesis factor 3 | 0.79 |
| GB45094 | golgin subfamily A member 4-like | 0.79 |
| GB48512 | phospholipase B1 | 0.78 |
| GB51332 | leucine-rich repeat-containing protein 40-like | 0.78 |
| GB45314 | cGMP-dependent 3',5'-cyclic phosphodiesterase-like | 0.77 |
| GB45696 | ETS-related transcription factor Elf-5-like | 0.76 |
| GB46986 | 39S ribosomal protein L46 | 0.76 |
| GB47813 | malonyl-CoA decarboxylase | 0.75 |
| GB47884 | TELO2-interacting protein 2-like | 0.75 |
| GB50627 | fatty acyl-CoA reductase | 0.75 |
| GB46265 | coiled-coil domain-containing protein 17 | 0.74 |
| GB44055 | NF-kappa-B inhibitor cactus 1 | 0.74 |
| GB51092 | conserved oligomeric Golgi complex subunit 3-like | 0.73 |
| GB54817 | muscle-specific protein 20 | 0.73 |
| GB42112 | cyclic AMP-dependent transcription factor ATF-6 alpha | 0.72 |
| GB43187 | uncharacterized | 0.72 |
| GB52314 | gamma-tubulin complex component 4 | 0.72 |
| GB49953 | vacuolar protein sorting-associated protein 45 | 0.71 |
| GB41444 | RNA cytidine acetyltransferase | 0.71 |
| GB55917 | kinetochore-associated protein 1 | 0.71 |
| GB42082 | adipocyte plasma membrane-associated protein | 0.71 |
| GB41294 | la-related protein 7 | 0.70 |
| GB55369 | WD repeat-containing protein CG11141 | 0.70 |
| GB53088 | cleft lip and palate transmembrane protein 1 homolog | 0.68 |
| GB51607 | monoacylglycerol lipase ABHD12 | 0.68 |
| GB47097 | transmembrane protein 35 | 0.67 |
| GB45913 | lethal(2)essential for life-like | 0.67 |
| GB41290 | myb-like protein D | 0.67 |
| GB50919 | uncharacterized | 0.67 |
| GB45113 | MIP18 family protein CG7949 | 0.66 |
| GB50955 | argonaute-2 | 0.66 |
| GB40967 | tyrosine hydroxylase | 0.66 |
| GB50106 | uncharacterized | 0.66 |
| GB51087 | pyridoxal kinase | 0.65 |
| GB40489 | NADH dehydrogenase | 0.65 |
| GB48946 | uncharacterized membrane protein | 0.65 |
| GB43264 | NADPH-dependent diflavin oxidoreductase 1 | 0.64 |
| GB44758 | eukaryotic translation initiation factor 2-alpha kinase | 0.62 |
| GB45973 | aromatic-L-amino-acid decarboxylase | 0.61 |
| GB41776 | regucalcin-like | 0.61 |
| GB40673 | lambda crystallin-like | 0.60 |
| GB44903 | calcineurin subunit B type 2 | 0.60 |
| GB49376 | F-box/LRR-repeat protein 3-like | 0.59 |
| GB42169 | MATH and LRR domain-containing protein PFE0570w-like | 0.59 |
| GB44894 | 28S ribosomal protein S2 | 0.58 |
| GB43112 | glycine-rich cell wall structural protein-like | 0.57 |
| GB42848 | deoxyribonuclease TATDN1 | 0.57 |
| GB47811 | uncharacterized | 0.57 |
| GB47596 | general transcription factor 3C polypeptide 1-like | 0.56 |
| GB54959 | GON-4-like | 0.56 |
| GB44506 | digestive organ expansion factor homolog | 0.55 |
| GB55967 | ribonuclease P protein subunit p40-like | 0.54 |
| GB43195 | uncharacterized | 0.52 |
| GB49607 | lysosome-associated membrane glycoprotein 1 | 0.52 |
| GB45260 | vacuolar protein sorting-associated protein 13A | 0.52 |
| GB42648 | ribophorin I | 0.52 |
| GB40654 | nuclear factor NF-kappa-B p100 subunit | 0.52 |
| GB43482 | ATP synthase subunit b | 0.48 |
| GB55456 | CWF19-like protein | 0.47 |
| GB52056 | insulin-like growth factor 2 mRNA-binding protein 1 | 0.47 |
| GB51299 | homeotic protein deformed | 0.45 |

a; Gene ID, BeeBase gene identifiers of the honey bee genome assembly 4.5^68, 69^

b; Gene description based on the National Center for Biotechnology Information^70^, and g:profiler search for cellular component gene ontology terms^71^

c; logFC; log_2_ fold change of the level of expression of each transcript from the differential expression analysis

**Table S2**. Significantly down-regulated DEGs in bees exposed to 1X10^-2^ng of clothianidin per µl compared to the bees exposed to 0 ng of clothianidin (0vs1X10^-2^) (Pearson pairwise comparison, p<0.05).

| **Gene ID^a^** | **Gene description^b^** | **LogFC^c^** |
| --- | --- | --- |
| GB49851 | uncharacterized | -6.42 |
| GB55213 | major royal jelly protein 7 | -2.13 |
| GB55212 | major royal jelly protein 2 | -1.53 |
| GB55211 | major royal jelly protein 2-like | -1.51 |
| GB49544 | vitellogenin | -1.35 |
| GB51373 | cell wall integrity and stress response component 1-like | -1.17 |
| GB55208 | major royal jelly protein 2 | -0.97 |
| GB41326 | venom acid phosphatase Acph-1-lik | -0.95 |
| GB51583 | kynurenine/alpha-aminoadipate aminotransferase | -0.91 |
| GB55209 | major royal jelly 5 | -0.84 |
| GB49543 | alanine--glyoxylate aminotransferase 2-like | -0.80 |
| GB54776 | atrial natriuretic peptide-converting enzyme | -0.76 |
| GB45365 | large neutral amino acids transporter small subunit 2 | -0.74 |
| GB52025 | membrane metallo-endopeptidase-like 1 | -0.71 |
| GB47165 | carboxypeptidase Q-like | -0.70 |
| GB53579 | glucosylceramidase 4 | -0.68 |
| GB51783 | carboxypeptidase Q-like | -0.68 |
| GB50115 | seminal fluid protein 53D artholog | -0.67 |
| GB47849 | pyrroline-5-carboxylate reductase 2 | -0.64 |
| GB43256 | ATP-binding cassette sub-family D member 1 | -0.63 |
| GB47148 | calcineurin-binding protein cabin-1-like | -0.61 |
| GB51487 | proton-coupled amino acid transporter 1-like | -0.60 |
| GB42311 | uncharacterized | -0.58 |
| GB48022 | henna | -0.58 |
| GB54316 | cardioacceleratory peptide receptor | -0.55 |
| GB51805 | proton-coupled amino acid transporter 4 | -0.54 |
| GB42508 | myosin 9 | -0.53 |
| GB46019 | la-related protein 1B | -0.50 |
| GB47736 | alkyldihydroxyacetonephosphate synthase | -0.50 |
| GB44808 | peroxidasin | -0.49 |
| GB44223 | lysosomal alpha-mannosidase-like | -0.48 |

a; Gene ID, BeeBase gene identifiers of the honey bee genome assembly 4.5^68,69^

b; Gene description based on the National Center for Biotechnology Information^70^, and g:profiler search for cellular component gene ontology terms^71^

c; logFC; log_2_ fold change of the level of expression of each transcript from the differential expression analysis

**Table S3.** Significantly up-regulated DEGs in bees exposed to 0 ng of clothianidin per µl compared to the bees parasitized with *V. destructor* (0vsVd) (Pearson pairwise comparison, p<0.05).

| **Gene ID^a^** | **Gene description^b^** | **LogFC^c^** |
| --- | --- | --- |
| GB50559 | uncharacterized membrane protein | 8.26 |
| GB51461 | uncharacterized | 6.87 |
| GB41338 | venom acid phosphatase | 4.90 |
| GB45796 | major royal jelly protein 3-like | 2.89 |
| GB55204 | major royal jelly protein 3 | 2.87 |
| GB55728 | CUGBP Elav-like family member 4 | 2.48 |
| GB54460 | UDP-glucuronosyltransferase 2B15-like | 2.44 |
| GB43510 | pancreatic triacylglycerol lipase-like | 2.13 |
| GB41217 | uncharacterized | 2.08 |
| GB49328 | fatty acyl-CoA reductase | 1.98 |
| GB45986 | scavenger receptor class B member 1 | 1.90 |
| GB51816 | glucose dehydrogenase | 1.87 |
| GB44120 | venom serine protease 34 | 1.85 |
| GB51815 | glucose dehydrogenase | 1.66 |
| GB51223 | hymenoptaecin | 1.65 |
| GB41817 | uncharacterized | 1.63 |
| GB41212 | laccase-5-like | 1.59 |
| GB47318 | abeacin | 1.53 |
| GB53516 | fatty acyl-CoA reductase | 1.44 |
| GB51874 | SLIT-ROBO Rho GTPase-activating protein 1-like | 1.41 |
| GB40619 | troponin C type IIIa | 1.37 |
| GB42593 | kinesin 9 | 1.34 |
| GB52864 | uncharacterized membrane protein | 1.34 |
| GB47970 | alpha-aminoadipic semialdehyde synthase | 1.32 |
| GB52056 | insulin-like growth factor 2 mRNA-binding protein 1 | 1.32 |
| GB50610 | repetitive proline-rich cell wall protein 2-like | 1.28 |
| GB53119 | apidermin 2 | 1.21 |
| GB49219 | armadillo repeat-containing protein 4 | 1.21 |
| GB43571 | esterase A2 | 1.18 |
| GB49394 | laccase-like | 1.16 |
| GB55273 | protein diaphanous | 1.14 |
| GB50545 | photoreceptor outer segment membrane glycoprotein 2-like | 1.11 |
| GB43005 | glucose dehydrogenase | 1.10 |
| GB51613 | COMM domain-containing protein 10 | 1.08 |
| GB42621 | fibroin heavy chain | 1.08 |
| GB51211 | neither inactivation nor afterpotential protein G-like | 1.08 |
| GB51299 | homeotic protein deformed | 1.06 |
| GB50627 | fatty acyl-CoA reductase | 1.06 |
| GB44500 | golgin subfamily A member 6-like protein 22 | 1.05 |
| GB53028 | laccase-1-like | 1.05 |
| GB46230 | odorant binding protein 21 | 1.03 |
| GB54226 | unconventional myosin-IXb | 0.99 |
| GB54817 | muscle-specific protein 20 | 0.99 |
| GB53116 | flocculation protein FLO11-like | 0.99 |
| GB46302 | 1-phosphatidylinositol 4,5-bisphosphate phosphodiesterase-like | 0.98 |
| GB47947 | titin homolog | 0.96 |
| GB55707 | inositol monophosphatase 2-like | 0.93 |
| GB49173 | 4-aminobutyrate aminotransferase, mitochondrial-like | 0.92 |
| GB50962 | POU domain protein CF1A | 0.92 |
| GB43672 | transient receptor potential-gamma protein-like | 0.91 |
| GB50123 | myophilin | 0.91 |
| GB52785 | carotenoid isomerooxygenase | 0.90 |
| GB42792 | uncharacterized | 0.90 |
| GB51790 | protein scarlet | 0.89 |
| GB41643 | blue-sensitive opsin | 0.88 |
| GB55196 | homeobox protein caupolican-like | 0.87 |
| GB51797 | ras-related protein Rab-32 | 0.85 |
| GB43788 | enhancer of split mbeta protein-like | 0.85 |
| GB50098 | arrestin homolog | 0.83 |
| GB54239 | zinc finger protein 853 | 0.83 |
| GB54097 | malvolio | 0.83 |
| GB50479 | uncharacterized | 0.82 |
| GB51515 | ras-responsive element-binding protein 1-like | 0.82 |
| GB44548 | glucose dehydrogenase | 0.81 |
| GB51189 | chaoptin | 0.81 |
| GB54118 | zinc finger protein rotund | 0.80 |
| GB52428 | uncharacterized | 0.79 |
| GB46301 | 1-phosphatidylinositol 4,5-bisphosphate phosphodiesterase-like | 0.79 |
| GB42673 | retinol dehydrogenase 10-A-like | 0.78 |
| GB40139 | peptidyl-prolyl cis-trans isomerase A2-like | 0.78 |
| GB50095 | MORN repeat-containing protein 4 | 0.76 |
| GB47942 | transient-receptor-potential-like protein | 0.76 |
| GB47990 | tropomyosin-1 | 0.75 |
| GB42178 | extra macrochaetae | 0.75 |
| GB44987 | vesicular glutamate transporter 2.1 | 0.72 |
| GB43052 | paramyosin, long form-like | 0.72 |
| GB40492 | 60S ribosomal protein L37 | 0.71 |
| GB41203 | cuticular protein analogous to peritrophins 3-C | 0.71 |
| GB42794 | circadian clock-controlled protein-like | 0.71 |
| GB43087 | uncharacterized membrane protein | 0.69 |
| GB46422 | proton-coupled amino acid transporter 1 | 0.69 |
| GB46612 | la-related protein 6 | 0.68 |
| GB47799 | protein hairy | 0.67 |
| GB43053 | paramyosin | 0.67 |
| GB51369 | ultraviolet-sensitive opsin | 0.64 |
| GB54611 | valbumin-related protein X | 0.64 |
| GB40673 | lambda crystallin-like protein | 0.63 |
| GB51653 | myosin heavy chain, muscle | 0.63 |

a; Gene ID, BeeBase gene identifiers of the honey bee genome assembly 4.5^68,69^

b; Gene description based on the National Center for Biotechnology Information^70^, and g:profiler search for cellular component gene ontology terms^71^

c; logFC; log_2_ fold change of the level of expression of each transcript from the differential expression analysis

**Table S4.** Significantly down-regulated DEGs in bees exposed to 0 ng of clothianidin per µl compared to the bees parasitized with *V. destructor* (0vsVd) (Pearson pairwise comparison, p<0.05).

| **Gene ID^a^** | **Gene description^b^** | **LogFC^c^** |
| --- | --- | --- |
| GB48843 | uncharacterized | -7.63 |
| GB48862 | cuticle protein 18.7-like | -7.15 |
| GB55029 | uncharacterized | -4.13 |
| GB46223 | odorant binding protein 14 | -4.03 |
| GB47830 | uncharacterized | -3.90 |
| GB48079 | trypsin alpha-3 | -3.84 |
| GB43688 | uncharacterized | -3.78 |
| GB43739 | carboxypeptidase B-like | -3.57 |
| GB50761 | chymotrypsin-1 | -3.37 |
| GB43690 | uncharacterized | -3.30 |
| GB48841 | cuticle protein 18.7-like | -3.30 |
| GB40136 | transmembrane protease serine 11B-like protein | -3.24 |
| GB42053 | epididymal secretory protein E1-like | -3.17 |
| GB50823 | protein trapped in endoderm-1 | -3.07 |
| GB44552 | flightin | -3.02 |
| GB44007 | BTB/POZ domain-containing protein 17 | -2.89 |
| GB44112 | melittin | -2.77 |
| GB55207 | major royal jelly protein 6 | -2.77 |
| GB42426 | puromycin-sensitive aminopeptidase-like protein | -2.75 |
| GB42434 | chitinase-3-like protein 1 | -2.65 |
| GB44100 | vegetative cell wall protein gp1-like | -2.62 |
| GB48969 | uncharacterized | -2.50 |
| GB44006 | alkylglycerol monooxygenase-like | -2.48 |
| GB52667 | monocarboxylate transporter 9-like | -2.42 |
| GB46286 | zinc carboxypeptidase-like | -2.41 |
| GB55263 | fatty acyl-CoA reductase | -2.38 |
| GB47563 | leucine-rich repeat-containing protein 70-like | -2.33 |
| GB52919 | uncharacterized | -2.31 |
| GB42427 | uncharacterized membrane protein | -2.29 |
| GB53978 | nodulin-75-like | -2.25 |
| GB53887 | uncharacterized | -2.11 |
| GB46587 | salivary secreted peptide | -2.09 |
| GB54782 | host cell factor 2-like | -2.07 |
| GB53911 | peritrophin-1-like | -2.00 |
| GB50236 | cuticular protein 14 | -1.91 |
| GB49854 | alpha-amylase | -1.89 |
| GB49811 | leucine-rich repeat-containing protein DDB_G0290503 | -1.86 |
| GB55208 | major royal jelly protein 5 | -1.64 |
| GB55209 | major royal jelly protein 5 | -1.51 |
| GB40299 | cuticular protein 5 | -1.50 |
| GB54549 | alpha-glucosidase | -1.38 |
| GB42468 | phospholipase B1, membrane-associated-like | -1.28 |
| GB55436 | uncharacterized | -1.16 |
| GB45073 | fibrillin-2-like | -1.13 |
| GB53200 | dnaJ homolog subfamily C member 18-like | -1.09 |
| GB51356 | cytochrome P450 4G11 | -1.03 |
| GB55170 | uncharacterized | -1.03 |
| GB50413 | protein TBRG4 | -1.00 |
| GB53579 | glucosylceramidase 4 | -0.95 |
| GB54170 | sodium-independent sulfate anion transporter | -0.93 |
| GB50118 | chymotrypsin inhibitor | -0.91 |
| GB48405 | 28S ribosomal protein S18b, mitochondrial | -0.89 |
| GB44225 | uncharacterized | -0.84 |
| GB43823 | chemosensory protein 1 | -0.81 |
| GB47506 | histone H1-like | -0.80 |
| GB55212 | major royal jelly protein 2 | -0.80 |
| GB41972 | monocarboxylate transporter 13-like | -0.79 |
| GB45495 | heat shock protein 83 | -0.77 |
| GB44427 | variant-silencing SET domain-containing protein-like | -0.76 |
| GB45194 | tyrosine-protein kinase transmembrane receptor Ror2 | -0.76 |
| GB54918 | GABA neurotransmitter transporter-1A | -0.74 |
| GB45968 | collagen alpha-1(IV) chain | -0.74 |
| GB52245 | speckle targeted PIP5K1A-regulated poly(A) | -0.74 |
| GB55889 | matrix metalloproteinase-14 | -0.74 |
| GB41284 | waprin-Thr1 | -0.73 |
| GB40021 | serine/threonine-protein kinase clkA | -0.69 |
| GB42964 | beta-1,3-glucosyltransferase | -0.68 |
| GB54602 | gamma-tubulin complex component 5 | -0.68 |
| GB55149 | uncharacterized membrane protein | -0.67 |
| GB42236 | patched domain-containing protein 3-like | -0.67 |
| GB46297 | cuticular protein 14 | -0.67 |
| GB52988 | uncharacterized | -0.67 |
| GB47805 | peptidoglycan recognition protein S2 | -0.65 |
| GB45910 | protein lethal(2)essential for life-like | -0.64 |
| GB41332 | actin | -0.62 |
| GB47459 | uncaracterized | -0.61 |
| GB45404 | innexin 1 | -0.60 |
| GB40746 | peptidyl-prolyl cis-trans isomerase FKBP4 | -0.60 |

a; Gene ID, BeeBase gene identifiers of the honey bee genome assembly 4.5^68,69^

b; Gene description based on the National Center for Biotechnology Information^70^, and g:profiler search for cellular component gene ontology terms^71^

c; logFC; log_2_ fold change of the level of expression of each transcript from the differential expression analysis

**Table S5.** Significantly up-regulated DEGs in bees exposed to 1X10^-2^ ng of clothianidin per µl plus *V. destructor* compared to the bees exposed to 0 ng of clothianidin per µl (0vs0.34+Vd) (Pearson pairwise comparison, p<0.05).

| **Gene ID^a^** | **Gene description^b^** | **LogFC^c^** |
| --- | --- | --- |
| GB47618 | defensin 2 | 6.87 |
| GB45796 | major royal jelly protein 3-like | 4.39 |
| GB54247 | uncharacterized | 3.69 |
| GB54400 | elongation of very long chain fatty acids protein | 3.69 |
| GB55204 | major royal jelly protein 3 | 3.68 |
| GB41338 | venom acid phosphatase | 3.34 |
| GB50559 | uncharacterized membrane protein | 2.90 |
| GB48803 | uncharacterized | 2.46 |
| GB43510 | pancreatic triacylglycerol lipasa-like | 1.86 |
| GB45986 | scavanger seceptor class B member 1 | 1.83 |
| GB42621 | fibroin heavy chain | 1.60 |
| GB44120 | venom acid protease 34 | 1.55 |
| GB53516 | fatty acil-CoA reductase | 1.47 |
| GB47318 | abeacin | 1.41 |
| GB50225 | WD repeat-containing 96-like | 1.41 |
| GB42800 | uncharacterized | 1.40 |
| GB51223 | hymenoptaecin | 1.39 |
| GB52775 | hyaluronoglucosaminidase | 1.36 |
| GB50005 | Kazal-type serine protease inhibitor | 1.31 |
| GB53028 | laccase-1 like | 1.31 |
| GB41212 | laccase-5-like | 1.28 |
| GB52056 | insulin-like growth factor 2 mRNA-binding protein 1 | 1.28 |
| GB50933 | GATA-binding factor A | 1.21 |
| GB42300 | uncharacterized | 1.18 |
| GB50610 | repetitive proline-rich cell wall protein 2-like | 1.16 |
| GB46001 | uncharacterized | 1.16 |
| GB43005 | glucose dehydrogenase | 1.13 |
| GB50962 | POU domain protein CF1A | 1.10 |
| GB46364 | 15-hydroxyprostaglandin dehydrogenase [NAD(+)]-like | 1.03 |
| GB52428 | uncharacterized | 1.02 |
| GB50627 | fatty acil-CoA reductase | 1.00 |
| GB54611 | ovalbumin-related protein X | 0.98 |
| GB54097 | malvolio | 0.98 |
| GB41806 | calcyphosin-like | 0.96 |
| GB42593 | kinesin 9 | 0.95 |
| GB51613 | COMM domain-containing protein 10 | 0.93 |
| GB44548 | glucose dehydrogenase | 0.91 |
| GB51299 | homeotic protein deformed | 0.89 |
| GB44192 | leucine-rich repeat-containing protein 26-like | 0.86 |
| GB51874 | SLIT-ROBO Rho GTPase-activating protein 1-like | 0.85 |
| GB54226 | unconventional myosin-Ixb | 0.84 |
| GB55212 | major royal jelly protein 2 | 0.84 |
| GB44055 | NK-kappa-B inhibitor cactus 1 | 0.83 |
| GB43418 | uncharacterized | 0.81 |
| GB40673 | lambda crystallin-like protein | 0.80 |
| GB51188 | lysophospholipid ayltransferase 2 | 0.78 |
| GB53798 | esterase E4-like | 0.77 |
| GB50795 | transcription factor AP-2-epsilon | 0.76 |
| GB47059 | protein tramtrack, beta isoform | 0.76 |
| GB54817 | muscle-specific protein 20 | 0.74 |
| GB46956 | homeobox protein B-H2-like | 0.73 |
| GB55206 | major royal jelly protein 4 | 0.72 |
| GB51515 | ras-responsive element-binding protein 1-like | 0.72 |
| GB45696 | ETS-related transcription factor Elf-5-like | 0.70 |
| GB52958 | mab-21 | 0.70 |
| GB49376 | F-box/LRR-repeat protein 3-like | 0.68 |
| GB53503 | transcriptional regulator Myc-B | 0.65 |
| GB46422 | proton-coupled amino acid transporter 1 | 0.65 |
| GB50931 | box A-binding factor-like | 0.64 |
| GB54595 | histone demethylase UTY | 0.62 |
| GB49601 | protein bark beetle | 0.60 |
| GB51809 | max-binding protein MNT | 0.60 |

a; Gene ID, BeeBase gene identifiers of the honey bee genome assembly 4.5^68,69^

b; Gene description based on the National Center for Biotechnology Information^70^, and g:profiler search for cellular component gene ontology terms^71^

c; logFC; log_2_ fold change of the level of expression of each transcript from the differential expression analysis

**Table S6.** Significantly down-regulated DEGs in bees exposed to 1X10^-2^ ng of clothianidin per µl plus *V. destructor* compared to the bees exposed to 0 ng of clothianidin per µl (0vs1X10^-2^ +Vd) (Pearson pairwise comparison, p<0.05).

| **Gene ID^a^** | **Gene description^b^** | **LogFC^c^** |
| --- | --- | --- |
| GB48843 | uncharacterized | -7.58 |
| GB48862 | cuticle protein 18.7-like | -6.73 |
| GB48432 | transmembrane protein 223 | -4.22 |
| GB50823 | trapped in endoderm-1 | -2.98 |
| GB48841 | cuticle protein 18.7-like | -2.96 |
| GB43739 | carboxypeptidase B-like | -2.59 |
| GB44007 | BTB/POZ domain-containing protein 17 | -2.35 |
| GB48969 | uncharacterized | -2.33 |
| GB42427 | uncharacterized | -2.09 |
| GB44112 | melitin | -2.09 |
| GB55915 | kinase epsilon | -2.02 |
| GB55207 | major royal jelly protein 6 | -1.95 |
| GB42426 | puromycin-sensitive aminopeptidase-like | -1.93 |
| GB54782 | host cell factor 2-like | -1.93 |
| GB50761 | chymotrypsin-1 | -1.60 |
| GB42053 | epididymal secretory protein E1-like | -1.58 |
| GB46595 | uncharacterized | -1.49 |
| GB54486 | myrosinase 1-like | -1.48 |
| GB45495 | heat shock protein 83 | -1.48 |
| GB50236 | cuticular protein CPF | -1.38 |
| GB50609 | heat shock protein Hsp70Ab-like | -1.34 |
| GB53200 | dnaJ homolog subfamily C member 18-like | -1.34 |
| GB50413 | TBRG4 | -1.30 |
| GB47082 | DALR anticodon-binding domain-containing protein 3-like | -1.27 |
| GB41809 | NADH dehydrogenase [ubiquinone] 1 alpha subcomplex assembly factor 3 | -1.21 |
| GB54572 | ribonuclease P protein subunit p30-like | -1.15 |
| GB40299 | cuticular protein 5 | -1.13 |
| GB53978 | early nodulin-75-like | -1.13 |
| GB42468 | phospholipase B1, membrane-associated-like | -1.08 |
| GB42745 | uncharacterized | -1.07 |
| GB55209 | major roya jelly protein 5 | -1.05 |
| GB42111 | uncharacterized | -1.05 |
| GB49250 | heme oxygenase | -0.94 |
| GB52245 | speckle targeted PIP5K1A-regulated poly(A) polymerase-like | -0.92 |
| GB50033 | COMM domain-containing protein 2 | -0.91 |
| GB40746 | peptidyl-prolyl cis-trans isomerase FKBP4 | -0.90 |
| GB55208 | major royal jelly protein 5 | -0.90 |
| GB54418 | uncharacterized | -0.89 |
| GB52650 | uncharacterized | -0.86 |
| GB43946 | uncharacterized | -0.85 |
| GB55149 | uncharacterized membrane protein | -0.85 |
| GB46774 | dnaJ protein homolog 1 | -0.84 |
| GB42419 | RNA polymerase II subunit B1 CTD phosphatase Rpap2 | -0.82 |
| GB42959 | CROWDED NUCLEI 3-like | -0.80 |
| GB44427 | ariant-silencing SET domain-containing protein-like | -0.78 |
| GB46297 | cuticular protein 14 | -0.76 |
| GB42964 | beta-1,3-glucosyltransferase | -0.72 |
| GB52988 | uncharacterized | -0.68 |
| GB47177 | ATP-dependent RNA helicase | -0.68 |
| GB54602 | gamma-tubulin complex component 5 | -0.68 |
| GB45404 | innexin 1 | -0.67 |
| GB45228 | chondroitin sulfate synthase 2 | -0.67 |
| GB54170 | sodium-independent sulfate anion transporter | -0.66 |
| GB47546 | apidaecin 1 | -0.66 |
| GB44298 | enoyl-CoA delta isomerase 1, mitochondrial-like | -0.65 |
| GB55917 | kinetochore-associated protein 1 | -0.62 |

a; Gene ID, BeeBase gene identifiers of the honey bee genome assembly 4.5^68,69^

b; Gene description based on the National Center for Biotechnology Information^70^, and g:profiler search for cellular component gene ontology terms^71^

c; logFC; log_2_ fold change of the level of expression of each transcript from the differential expression analysis

**Table S7**. Gene IDs s in common between the pairwise comparisons of 0 ng of clothianidin vs 1X10^-2^ ng/µl of clothianidin (0vs1X10^-2^), 0 ng of clothianidin vs *V. destructor* (0vsVd) and 0 ng of clothianidin vs 1X10^-2^ ng/µl of clothianidin plus *V. destructor* (0vs1X10^-2^ +Vd).

| **Pairwise comparisons** | **Up-regulated** | **Down-regulated** |
| --- | --- | --- |
| 0vs1X10^-2^, 0vsVd, 0vs1X10^-2^+Vd | GB51299, GB43510, GB54611, GB54097, GB42621, GB50627, GB54817, GB52056, GB41338, GB51223, GB53028, GB40673 | GB55208, GB55209 |
| 0vs1X10^-2^, 0vsVd |  | GB55212, GB53579, |
| 0vs1X10^-2^, 0vs1X10^-2^+Vd | GB41806, GB54400, GB44055, GB50005, GB45696, GB53798, GB49376, GB47618, GB43418 | GB44007, GB42964, GB53200, GB44112, GB42053, GB48843, GB54602, GB50761, GB48969, GB40299, GB44427, GB46297, GB50823, GB55149, GB48841, GB55207, GB42426, GB52245, GB43739, GB48862, GB42427, GB50413, GB52988, GB42468, GB45404, GB53978, GB50236, GB40746, GB54170, GB45495, GB54782 |
| 0vs1X10^-2^+Vd, 0vsVd | GB54226, GB45796, GB51613, GB50559, GB46422, GB50962, GB42593, GB44548, GB53516, GB50610, GB55204, GB47318, GB45986, GB51515, GB43005, GB41212, GB44120, GB52428, GB51874 | GB44007, GB42964, GB53200, GB44112, GB42053, GB48843, GB54602, GB50761, GB48969, GB40299, GB44427, GB46297, GB50823, GB55149, GB48841, GB55207, GB42426, GB52245, GB43739, GB48862, GB42427, GB50413, GB52988, GB42468, GB45404, GB53978, GB50236, GB40746, GB54170, GB45495,  GB54782 |
| 0vs1X10^-2^ | GB40890, GB43815, GB47347, GB49086, GB48954, GB41925, GB51990, GB41026, GB44068, GB48820, GB51606, GB40810, GB41490, GB46265, GB42848, GB52190, GB42964, GB41078, GB43639, GB50253, GB42754, GB47082, GB45094, GB41444, GB41173, GB55436, GB54959, GB55111, GB44455, GB47595, GB44901, GB40903, GB43540, GB42376, GB53876, GB43169, GB47177, GB50641, GB43822, GB44144, GB40800, GB47097, GB45861, GB41973, GB52314, GB43946, GB53088, GB51202, GB45697, GB43784, GB48578, GB49234, GB54955, GB42082, GB43193, GB45085, GB45808, GB52744, GB40562, GB50346, GB40782, GB49956, GB44506, GB41118, GB46595, GB52673, GB40489, GB49524, GB46534, GB52470, GB46881, GB51132, GB55191, GB47459, GB52888, GB49414, GB42615, GB55369, GB55456, GB55149, GB52011, GB47108, GB54469, GB50883, GB48373, GB51087, GB40727, GB55917, GB42050, GB47813, GB52245, GB42958, GB50550, GB51092, GB49607, GB45422, GB48379, GB51719, GB45314, GB40967, GB52805, GB54655, GB41657, GB54212, GB42650, GB43112, GB47546, GB49371, GB54960, GB48408, GB45355, GB47884, GB48581, GB42419, GB51061, GB42919, GB41976, GB55810, GB52638, GB42081, GB45125, GB51607, GB51272, GB42383, GB44758, GB42631, GB52244, GB41339, GB45366, GB54091, GB52685, GB46986, GB54572, GB49953, GB45210, GB55298, GB51602, GB40884, GB50955, GB45913, GB50213, GB43783, GB47207, GB50423, GB53200, GB55157, GB48484, GB47538, GB55457, GB45228, GB43187, GB41099, GB44571, GB49582, GB46267, GB43195, GB42112, GB43485, GB44504, GB54602, GB50033, GB46706, GB48946, GB50343, GB51338, GB44264, GB54293, GB48923, GB52577, GB47337, GB48922, GB50420, GB51627, GB43134, GB44894,GB55110, GB41977, GB51535, GB45260, GB50323, GB45113, GB54050, GB53663, GB41153, GB46182, GB55156, GB41290, GB53649, GB48835, GB50039, GB51367, GB55826, GB42704, GB46563, GB50352, GB45973, GB41809, GB55979, GB47315, GB42653, GB55967, GB47596, GB50919, GB51332, GB50106, GB55939, GB54420, GB44514, GB54108, GB42648, GB52235, GB44903, GB48512, GB41776, GB43482, GB43901, GB51637, GB41294, GB43561, GB54610, GB49642, GB50413, GB44641, GB42380, GB52560, GB53193, GB51001, GB42169, GB54954, GB43900, GB42015, GB51601, GB45312, GB45653, GB42083, GB47487, GB40546, GB47811, GB49385, GB40654, GB43264, GB51306, GB41142, GB45122, GB47571, GB48084, | GB51487, GB49543, GB49851, GB48022, GB47165, GB51583, GB43256, GB52025, GB47849, GB55211, GB47148, GB47736, GB51783, GB46019, GB54776, GB50115, GB49544, GB55213, GB42311, GB45365, GB41326, GB44808, GB54316, GB44223, GB42508, GB51373, GB51805 |
| 0vsVd | GB46230, GB40139, GB49394, GB55728, GB51790, GB41643, GB46302, GB50123, GB51461, GB51815, GB42673, GB53119, GB52864, GB46612, GB50545, GB47947, GB42792, GB44987, GB47942, GB51369, GB40619, GB49219, GB50479, GB55196, GB46301, GB50095, GB42794, GB52785, GB47990, GB53116, GB54460, GB50098, GB55707, GB43672, GB47799, GB42178, GB51653, GB49328, GB43053, GB41817, GB51797, GB43087, GB43571, GB51816, GB54118, GB43052, GB47970, GB51211, GB41217, GB43788, GB54239, GB51189, GB44500, GB40492, GB41203, GB49173, GB55273 | GB45073, GB43823, GB40136, GB43690, GB41332, GB54918, GB55436, GB47830, GB53887, GB45910, GB48405, GB55170, GB55029, GB45968, GB46286, GB46223, GB41284, GB45194, GB46587, GB52667, GB40021, GB55263, GB47459, GB48079, GB54549, GB42434, GB51356, GB55889, GB43688, GB44552, GB50118, GB47563, GB44006, GB49811, GB53911, GB49854, GB44100, GB52919, GB47506, GB42236, GB41972, GB44225, GB47805 |
| 0vs1X10^-2^+Vd | GB46956, GB47059, GB50933, GB42300, GB55212, GB50225, GB50795, GB53503, GB52958, GB42800, GB51809, GB50931, GB46364, GB55206, GB46001, GB52775, GB54595, GB49601, GB48803, GB44192, GB54247,  GB51188 | GB55915, GB49250, GB48432, GB54572, GB42111, GB47082, GB45228, GB42959, GB47177, GB50033, GB43946, GB46595, GB44298, GB46774, GB41809, GB55917, GB54486, GB54418, GB50609, GB52650, GB47546, GB42419, GB42745 |

**Table S8**. KEGG pathways analysis of the DEGs (up-regulated) between the bees treated with 0 ng and 1X10^-2^ ng of clothianidin per µl (0vs1X10^-2^).

| **Gene ID^a^** | **Gene description^b^** | **Biological pathway^c^** |
| --- | --- | --- |
| GB43169 | phosphatidylinositol N-acetylglucosaminyltransferase subunit C | metabolic pathway (ko01100); glycosylphosohatidylinositol (GPI)-anchor biosynthesis (ko00563) |
| GB52244 | telomerase reverse transcriptase | HTLV-I infection (ko05166); human papillomavirus infection (ko05165) |
| GB55110 | 7-methylguanosine phosphate-specific 5'-nucleotidase | metabolic pathway (ko01100); biosynthesis of secondary metabolites (ko01110); purine metabolism (ko00230); purymidine metabolism (ko00240); nicotinate and nictinamide metabolism (ko00760) |
| GB47618 | defensin 2 | Toll and Imd signaling pathway (ko04624) |
| GB45422 | transmembrane protein 231 | ubiquitin mediated proteolysis (ko04120) |
| GB48084 | ethanolamine kinase 1 | metabolic pathway (ko01100); glycerophospholipid metabolism (ko00564) |
| GB47487 | oligoribonuclease | ribosome biogenesis in eukaryotes (ko03008) |
| GB52470 | ribonuclease H2 subunit C | DNA replication (ko03030) |
| GB42615 | uncharacterized | ribosome (ko03010) |
| GB45210 | translocon-associated protein subunit gamma-like | protein processing in endoplasmic reticulum (ko014141) |
| GB42964 | beta-1,3-glucosyltransferase | other types of O-glycan biosynthesis (ko00514) |
| GB49371 | NADH-cytochrome b5 reductase-like | amino sugar and nucleotide sugar metabolism (ko00520) |
| GB52805 | phosphatidylinositol N-acetylglucosaminyltransferase subunit Q | metabolic pathway (ko01100); glycosylphosohatidylinositol (GPI)-anchor biosynthesis (ko00563) |
| GB48835 | EKC/KEOPS complex subunit TPRKB-like | inole alkaloid biosynthesis (ko00950) |
| GB50343 | ribonuclease P protein subunit p29 | RNA transport (ko03013); ribosome biogenesis in eukaryotes (ko03008) |
| GB47207 | thioredoxin | NOD-like receptor signaling pathway (ko04621); fluid shear stress and atherosclerosis (ko05418) |
| GB50346 | translation initiation factor eIF-2B subunit epsilon | RNA transport (ko03013) |
| GB41153 | peroxisomal membrane protein PEX16 | peroxisome (ko04146) |
| GB42653 | glycogenin- | metabolic pathway (ko01100); starch and sucrose metabolism (ko00500) |
| GB54050 | chitobiosyldiphosphodolichol beta-mannosyltransferase-like | metabolic pathway (ko01100); N-glycan biosynthesis (ko00510); various types of N-glycan biosynthesis (ko00513) |
| GB48578 | lethal(2)neighbour of Tid | metabolic pathway (ko01100); N-glycan biosynthesis (ko00510); various types of N-glycan biosynthesis (ko00513) |
| GB50323 | phosphatidylinositol glycan anchor biosynthesis class U | metabolic pathway (ko01100); glycosylphosohatidylinositol (GPI)-anchor biosynthesis (ko00563) |
| GB49524 | GPI mannosyltransferase 3 | metabolic pathway (ko01100); glycosylphosohatidylinositol (GPI)-anchor biosynthesis (ko00563) |
| GB54960 | oligoribonuclease | ribosome biogenesis in eukaryotes (ko03008) |
| GB43815 | 39S ribosomal protein L23 | ribosome (ko03010) |
| GB51627 | dehydrodolichyl diphosphate syntase complex subunit DHDDS (LOC551358), transcript variant X3 | biosynthesis of secondary metabolites (ko01110); terpenoid backbone biosynthesis (ko00900) |
| GB42919 | Fanconi anemia group D2 protein | Fanconi anemia pathway (ko03460) |
| GB54097 | malvolio | lysosome (ko04142); ferropoptosis (ko04216); mineral absorption (ko04978) |
| GB44641 | F-box/WD repeat-containing protein 9 | vitamin digestion and absorption (ko04977) |
| GB51719 | ribonuclease Z | RNA transport (ko03013) |
| GB48923 | microfibrillar-associated protein 1 | microRNAs in cancer (ko05206) |
| GB49582 | double-strand break repair protein MRE11 | homologous recombination (ko03440); non-homologous end-joining (ko034500); cellular senescence (ko0418) |
| GB50352 | glutathione synthetase | metabolic pathway (ko01100); cysteine and methionine metabolism (ko00270); glutathione metabolism (ko00480); ferropoptosis (ko04216) |
| GB41977 | m7GpppX diphosphatase | RNA degradation (ko03018) |
| GB44455 | uncharacterized | Toll and Imd signaling pathway (ko04624) |
| GB42650 | GPI mannosyltransferase 4 | glycosylphosohatidylinositol (GPI)-anchor biosynthesis (ko00563) |
| GB45125 | mRNA cap guanine-N7 methyltransferase | mRNA surveillance pathway (ko03015) |
| GB47571 | dihydroxyacetone phosphate acyltransferase | glycophospholipid metabolism (ko00564); peroxisome (ko04146) |
| GB49086 | folylpolyglutamate synthase | metabolic pathway (ko01100); folate biosynthesis (ko00790) |
| GB45228 | chondroitin sulfate synthase 2 | metabolic pathway (ko01100); glycosaminoglycan biosynthesis-chondroitin sulfte/ermatal sulfate (ko00532) |
| GB41142 | dolichyl pyrophosphate Glc1Man9GlcNAc2 alpha-1,3-glucosyltransferase | metabolic pathway (ko01100); N-glycan biosynthesis (ko00510) |
| GB50420 | TELO2-interacting protein 1 homolog | mTOR signaling pathway (ko04150) |
| GB44144 | phenylalanine--tRNA ligase | aminoacyl-tRNA biosynthesis (ko00970) |
| GB40800 | signal peptidase complex subunit 3 | protein export (ko03060) |
| GB42081 | MATH and LRR domain-containing protein PFE0570w-like | metabolic pathway (ko01100); glycosylphosohatidylinositol (GPI)-anchor biosynthesis (ko00563) |
| GB43901 | DNA replication ATP-dependent helicase/nuclease DNA2 | DNA replication (ko03030) |
| GB50039 | methionine--tRNA ligas | selenocompound metabolism (ko00450); aminoacyl-tRNA biosynthesis (ko00970) |
| GB55979 | ribonuclease P protein subunit p40-like | RNA transport (ko03013); ribosome biogenesis in eukaryotes (ko03008) |
| GB49642 | DNA-dependent protein kinase catalytic subunit-like | non-homologous end-joining (ko034500); cell cycle (ko04110) |
| GB54108 | dual specificity protein phosphatase 3 | MAPK signaling pathway (ko04010) |
| GB43485 | peroxisomal biogenesis factor 3 | peroxisome (ko04146) |
| GB45314 | cGMP-dependent 3',5'-cyclic phosphodiesterase-like | purine metabolism (ko00230); cGMP-PKG signaling pathway (ko04022); aldosterone synthesis and secretion (ko04925); olfactory transduction (ko04740); morphine addiction (ko050352) |
| GB47813 | malonyl-CoA decarboxylase | metabolic pathway (ko01100); propanoate metabolism (ko00640); beta-alanine metabolism (ko00410); AMPK signaling pathway (ko04152); peroxisome (ko04146) |
| GB50627 | fatty acyl-CoA reductase | cutin, suberine and wax biosynthesis (ko0073); peroxisome (ko04146); longetivy regulating pathway-worm (ko04212) |
| GB42112 | cyclic AMP-dependent transcription factor ATF-6 alpha | protein processing in endoplasmic reticulum (ko014141); Alzheimer's disease (ko05010) |
| GB49953 | vacuolar protein sorting-associated protein 45 | endoytosis (ko04144); autophagy-yeast (ko04138) |
| GB41444 | RNA cytidine acetyltransferase | ribosome biogenesis in eukaryotes (ko03008) |
| GB45913 | lethal(2)essential for life-like | protein processing in endoplasmic reticulum (ko014141); longetivey regulating pathway-multiple species (ko04213) |
| GB45113 | MIP18 family protein CG7949 | protein export (ko03060); bacterial secretion system (ko03460); quorum sensing (ko02024) |
| GB50106 | uncharacterized | metabolic pathway (ko01100); oxidative phosphorylation (ko00190); phagosome (ko04145); lysosome (ko04142); rheumatoid arthritis (ko05323); Vibrio cholerae infection (ko05110); epithelial cell signlaing in Helicobacter pylori infection (ko05120); tuberculosis (ko05152); hepatitis B (ko05161) |
| GB51087 | pyridoxal kinase | metabolic pathway (ko01100); vitamin B6 metabolism (ko00750) |
| GB40489 | NADH dehydrogenase | metabolic pathway (ko01100); oxidative phosphorylation (ko00190); retrograde andocannabinoid signaling (ko04723); Alzheimer's disease (ko05010); Parkinson's disease (ko05012); Huntington's disease (ko05016); non-alcoholic fatty liver disease (ko04932) |
| GB44758 | eukaryotic translation initiation factor 2-alpha kinase | protein processing in endoplasmic reticulum (ko014141); autophagy-animal (ko04140); mitophagy-animal (ko04137); apoptosis (ko0421); apoptosis-fly (ko04214); Alzheimer's disease (ko05010); non-alcoholic fatty liver disease (ko04932); measles (ko05162); influenza A (ko05164); hepatitis C (ko05160); herpes simplex infection (ko05168) |
| GB45973 | aromatic-L-amino-acid decarboxylase | metabolic pathway (ko01100); biosynthesis of secondary metabolites (ko01110); amphetamine addiction (ko05031); alcoholism (ko05034); histidine metabolism (ko00340); tyrosine metabolism (ko00350); phenylalanine metabolism (ko00360); tryptophan metabolism (ko00380); isoquinone alkaloid biosynthesis (ko00950); betalain biosynthesis (ko00965); dopaminergic synapse (ko04728); serotonergic synapse (ko04726); cocaine addiction (ko05030) |
| GB40673 | lambda crystallin-like | metabolic pathway (ko01100); pentose and gluconate interconversions (ko00040) |
| GB44903 | calcineurin subunit B type 2 | MAPK signaling pathway (ko04010); Wnt singaling pathway (ko04310); VEGF singlaing pathway (ko04370); Calcium signaling pathway (ko04020); cGMP-PKG signaling pathway (ko04022); oocyte meiosis (ko04114); cellular senescence (ko0418); natural killer cell mediated cytotoxicity (ko04650); T cell receptor signaling pathway (ko04660); Th1 and Th2 cell differentiation (ko04658); Th17 cell differentiation (ko04659); B cell receptor signaling pathway (ko04662); glucagon signaling pathway (ko04922); oxytocin singnaling pathway (ko04921); renin secretion (ko04924); glutamatergic synapse (ko04724); long-term potentiation (ko04720); axon guidance (ko04360); osteoclast differentiation (ko04380); Alzheimer's disease (ko05010); amphetamine addiction (ko05031); tuberculosis (ko05152); HTLV-I infection (ko05166); Kaposi's sarcoma-associated herpesvirus infection (ko05167); Epstein-Barr virus infection (ko05169) |
| GB49376 | F-box/LRR-repeat protein 3-like | circadian rhythm (ko04710) |
| GB49607 | lysosome-associated membrane glycoprotein 1 | phagosome (ko04145); lysosome (ko04142); autophagy-animal (ko04140); tuberculosis (ko05152) |
| GB40654 | nuclear factor NF-kappa-B p100 subunit | ras signaling pathway (ko04014); MAPK signaling pathway (ko04010); NF-kappa B signaling pathway (ko04064); TNF signaling pathway (ko04668); HIF-1 signaling pathway (ko04066); Shingolipid signaling pathway (ko04071); cAMP signaling pathway (ko04024); PI3K-Akt signaling pathway (ko04151); apoptosis (ko0421); cellular senescence (ko0418); Toll-loke receptor singaling pathway (ko04620); Toll and Imd signaling pathway (ko04624); NOD-like receptor signaling pathway (ko04621); RIG-I-like receptor signaling pathway (ko04622); cytosolic DNA-sensing pathway (ko04623); cytosolic DNA-sensing pathway (ko04623); T cell receptor signaling pathway (ko04660); Th1 and Th2 cell differentiation (ko04658); Th17 cell differentiation (ko02580); IL-17 singaling patway (ko04657); B cell receptor signaling pathway (ko04662); chemokine signaling pathway (ko04062); adipocytokine signaling pathway (ko04920); prolactin signaling pathway (ko04917); neurotrophic signlaing pathway (ko04722); osteoclast differentiation (ko04380); longetive regulating pathway (ko04211); pathways in cancer (ko05200); transcriptional misregulation in cancer (ko05202); microRNAs in cancer (ko05206); viral carcinogenesis (ko05203); pancreatic cancer (ko05212); acute myeloid leukemia (ko05221); chronic myeloid leukemia (ko05220); prostate cancer (ko05215); small cell lung cancer (ko05222); inflammatory bowel disease (ko05321); cocaine addiction (ko05030); fluid shear stress and atherosclerosis (ko05418); non-alcoholic fatty liver disease (ko04932); insuline resistance (ko04931); AGE-RAGE signaling pathway in diabetic complications (ko04933); epithelial cell signlaing in Helicobacter pylori infection (ko05120); Salmonella infection (ko05132); Shigellosis (ko05131); pertussis (ko05133); legionellosis (ko05134); tuberculosis (ko05152); HTLV-I infection (ko05166); measles (ko05162); influenza A (ko05164); hepatitis B (ko5160); hepatitis C (ko05160); herpes simplex infection (ko05168)d; Kaposi's sarcoma-associated herpesvirus infection (ko05167); Epstein-Barr virus infection (ko05169); human papillomavirus infection (ko05165); amoebiasis (ko05146); toxoplasmosis (ko05145); leishmaniasis (ko05140) |
| GB43482 | ATP synthase subunit b | metabolic pathway (ko01100); oxidative phosphorylation (ko00190); Alzheimer's disease (ko05010); Parkinson's disease (ko05012); Huntington's disease (ko05016) |
| GB52056 | insulin-like growth factor 2 mRNA-binding protein 1 | microRNAs in cancer (ko05206) |

a; Gene ID, BeeBase gene identifiers of the honey bee genome assembly 4.5^68,69^

b; Gene description based on the National Center for Biotechnology Information^70^, and g:profiler search for cellular component gene ontology terms^71^

c; Biological pathways and KEGG ontology identifiers (ko) identifiers from biological pathways based on KASS search^66^.

**Table S9.** KEGG pathways analysis of the DEGs (down-regulated) between the bees treated with 0 ng and 1X10^-2^ ng of clothianidin per µl (0vs1X10^-2^).

| **Gene ID^a^** | **Gene description^b^** | **Biological pathway^c^** |
| --- | --- | --- |
| GB51583 | kynurenine/alpha-aminoadipate aminotransferase | metabolic pathway (ko01100); biosynthesis of antibiotics (ko01130); 2-oxocarboxylic acid metabolism (ko01210); biosynthesis of amino acids (ko01230); lysine biosynthesis (ko00300); lysine degradation (ko000310); tryptophan metabolism (ko00380) |
| GB49543 | alanine--glyoxylate aminotransferase 2-like | metabolic pathway (ko01100); glycerophospholipid metabolism (ko00564) |
| GB53579 | putative glucosylceramidase 4 | metabolic pathway (ko01100); sphingolipid metabolism (ko00600); other glycan degradation (ko00511); lysosome (ko04142) |
| GB47849 | pyrroline-5-carboxylate reductase 2 | metabolic pathway (ko01100); biosynthesis of secondary metabolites (ko01110); biosynthesis of antibiotics (ko01130); biosynthesis of amino acids (ko01230); arginine and proline metabolism (ko00330) |
| GB43256 | ATP-binding cassette sub-family D member 1 | ABC transporters (ko02010); peroxisome (ko04146) |
| GB51487 | proton-coupled amino acid transporter 1-like | autophagy-yeast (ko04138) |
| GB48022 | henna | metabolic pathway (ko01100); biosynthesis of amino acids (ko01230); phenylalanine metabolism (ko00360); phenylalanine, tyrosine and tryptophan biosynthesis (ko00400) |
| GB51805 | proton-coupled amino acid transporter 4 | autophagy-yeast (ko04138) |
| GB47736 | alkyldihydroxyacetonephosphate synthase | metabolic pathway (ko01100); ether lipid metabolism (ko00565); peroxisome (ko04146) |
| GB44223 | lysosomal alpha-mannosidase-like | other glycan degradation (ko00511); lysosome (ko04142) |

a; Gene ID, BeeBase gene identifiers of the honey bee genome assembly 4.5^68,69^

b; Gene description based on the National Center for Biotechnology Information^70^, and g:profiler search for cellular component gene ontology terms^71^

c; Biological pathways and KEGG ontology identifiers (ko) identifiers from biological pathways based on KASS search^66^.

**Table S10.** KEGG pathways analysis of the DEGs (up-regulated) between the bees parasitized with *V. destructo*r compared to bees exposed to 0 ng of clothianidin (0vsVd).

| **Gene ID^a^** | **Gene description^b^** | **Biological pathway^c^** |
| --- | --- | --- |
| GB49328 | fatty acyl-CoA reductase | cutin, sunerine and wax biosynthesis (ko0073); peroxisome (ko04146); longevity regulating pathway-worm (ko04212) |
| GB53516 | fatty acyl-CoA reductase | cutin, sunerine and wax biosynthesis (ko0073); peroxisome (ko04146) |
| GB51874 | SLIT-ROBO Rho GTPase-activating protein 1-like | axon guidance (ko04360) |
| GB47970 | alpha-aminoadipic semialdehyde synthase | metabolic pathway (ko01100); biosynthesis of secondary metabolites (ko01110);biosynthesis of antibiotics (ko01130); lysine degradation (ko00310) |
| GB52056 | insulin-like growth factor 2 mRNA-binding protein 1 | microRNAs in cancer (ko05206) |
| GB50627 | putative fatty acyl-CoA reductase | cutin, sunerine and wax biosynthesis (ko0073); peroxisome (ko04146) |
| GB54226 | unconventional myosin-IXb | hippo signaling pathway-fly (ko04391) |
| GB46302 | 1-phosphatidylinositol 4,5-bisphosphate phosphodiesterase-like | metabolic pathway (ko01100); inositol phosphate metabolism (ko00562); rap1 signaling pathway (ko04015); Wnt signaling pathway (ko04310); apelin singaling pathway (ko04371); calcium singnaling pathway (ko04020); phosphatidylinositol signaling system (ko04070); phospholipase D signaling pathway (ko04072); sphingolipid signaling pathway (ko04071); cGMP-PKG signaling pathway (ko04022); Gap junction (ko04540); platelet activation (ko04611); NOD-like receptor singaling pathway (ko04621); chemokine signaling pathway (ko04062); insulin secretion (ko04911); glucagon singaling pathway (ko04922); GnRH signaling pathway (ko04912); estrogen signaling pathway (ko04915); oxytocin signaling pathway (ko04921); thyroid hormone synthesis (ko04918); thyroid hormone signaling pathway (ko04919); melanogenesis (ko04916); renin secretion (ko04924); aldosterone synthesis and secretion (ko04925); adrenergic signaling in cardiomyocytes (ko04261); vascular smooth muscle contraction (ko04270); salivary secretion (ko04970); gastric acid secretion (ko04971); pancreatic secretion (ko04972); endocrine and other factor-regulated calcium reabsorption (ko04961); glutamatergic synapse (ko04724); cholinergic synapse (ko04725); dopaminergic synapse (ko04728); serotonergic synapse (ko04726); long-term potentiation (ko04720); long-term depression (ko04730); retrograde endocannabinoid signaling (ko04723); phototransduction-fly (ko04745); inflammatory mediator regulation of TRP channels (ko04750); circadian entrainment (ko04713); pathways in cancer (ko05200); Alzheimer's disease (ko05010); Huntington's disease (ko05010); AGE-RAGE signaling pathway in diabetic complications (ko04933); amoebiasis (ko05146); Chagas disease (ko05142); African trypanosomiasis (ko05143) |
| GB55707 | inositol monophosphatase 2-like | metabolic pathway (ko01100); inositol phosphate metabolism (ko00562); streptomycin biosynthesis (ko00521); phosphatidylinositol signaling system (ko04070) |
| GB49173 | 4-aminobutyrate aminotransferase, mitochondrial-like | metabolic pathway (ko01100); microbial metabolism in diverse environments (ko01120); propanate metabolism (ko00640); butanoate metabolism (ko00650); alanine, aspartate and glutamate metabolism (ko00250); valine, leucine and isoleucine degradation (ko00280); beta-alanine metabolism (ko00410); GABAergic synapse (ko04727) |
| GB54097 | malvolio | lysosome (ko04142); ferroptosis (ko04216); mineral absorption (ko04978) |
| GB44548 | glucose dehydrogenase | metabolic pathway (ko01100); glycine, serine and threonine metabolism (ko00260) |
| GB47942 | transient-receptor-potential-like protein | phototransduction-fly (ko04745) |
| GB47990 | tropomyosin-1 | cardiac muscle contraction (ko04260); adrenergic signaling in cardiomyocytes (ko04261); microRNAs in cancer (ko05206); hypertrophic cardiomyopathy (ko05410) |
| GB42178 | extra macrochaetae | TGF-beta signaling pathway (ko04350) |
| GB40492 | 60S ribosomal protein L37 | ribosome (ko03010) |
| GB46422 | proton-coupled amino acid transporter 1 | autophagy-yeast (ko04138) |
| GB40673 | lambda crystallin-like protein | metabolic pathway (ko01100); pentose and glucuronate interconversions (ko0040) |
| GB51653 | myosin heavy chain, muscle | cardiac muscle contraction (ko04260); adrenergic signaling in cardiomyocytes (ko04261); viral myocarditis (ko05416) |

a; Gene ID, BeeBase gene identifiers of the honey bee genome assembly 4.5^68,69^

b; Gene description based on the National Center for Biotechnology Information^70^, and g:profiler search for cellular component gene ontology terms^71^

c; Biological pathways and KEGG ontology identifiers (ko) identifiers from biological pathways based on KASS search^66^.

**Table S11**. KEGG pathways analysis of the DEGs (down-regulated) between the bees parasitized with *V. destructo*r compared to bees exposed to 0 ng of clothianidin per µl (0vsVd).

| **Gene ID** | **Gene desciption** | **Biological pathway** |
| --- | --- | --- |
| GB48079 | trypsin alpha-3 | protein digestion and absorption (ko04974); influenza A (ko05164); nueroactive ligand-receptor interaction (ko04080); pancreatic secretion (ko04972) |
| GB42053 | epididymal secretory protein E1-like | lysosome (ko04142) |
| GB42434 | chitinase-3-like protein 1 | metabolic pathways (ko01100); amino sugar and nucleotide sugar metabolism (ko00520) |
| GB55263 | putative fatty acyl-CoA reductase | cutin, suberine and wax biosynthesis (ko00073); longevity regulation pathway-worm (ko04212); longevity regulating pathway-worm (ko04212) |
| GB49854 | alpha-amylase | metabolic pathways (ko01100); starch and sucrose metabolism (ko00500); carbohydrate digestion and abosrption (ko04973) |
| GB54549 | alpha-glucosidase | metabolic pathways (ko01100); starch and sucrose metabolism (ko00500); galactose metabolism (ko00052) |
| GB53579 | putative glucosylceramidase 4 | metabolic pathways (ko01100); lysosome (ko04142); other glucan degradation (ko00511); sphingolipid metabolism (ko00600) |
| GB48405 | 28S ribosomal protein S18b, mitochondrial | viral carcinogenesis (ko05203) |
| GB45495 | heat shock protein 83 | estrogen signaling pathway (ko04915); pathways in cancer (ko05200); protein processing in endoplasmic reticulum (ko04141); PI3K-Akt signaling pathway (ko04151); progesterone-mediated oocyte maturation (ko04914); fluid shear stress and atherosclerosis (ko05418); NOD-like receptor signaling pathway (ko04621); plant-pathogen interaction (ko04626); necroptosis (ko04217); IL-17 singaling pathway (ko04657); antigen processing and presentation (ko04612); Th17 cell differentiation (ko04659); prostate cancer (ko05215) |
| GB54918 | GABA neurotransmitter transporter-1A | GABAergic synapse (ko04727) |
| GB45968 | collagen alpha-1(IV) chain | PI3K-Akt signaling pathway (ko04151); protein digestion and absorption (ko04974); ECM-receptor interaction (ko04512); human papillomavirus infection (ko05165); amoebiasis (ko05146); small cell lung cancer (ko05222); AGE-RAGE signaling pathway in diabetic complications (ko04933); focal adhesion (ko04510) |
| GB55889 | matrix metalloproteinase-14 | TNF signaling pathway (ko04668); GnRH signaling pathway (ko04912) |
| GB42964 | beta-1,3-glucosyltransferase | other types of O-glycan biosynthesis (ko00514) |
| GB45910 | protein lethal(2)essential for life-like | protein processing in endoplasmic reticulum (ko04141); longevity regulating pathway-multiple species (ko04213) |
| GB41332 | actin | metabolic pathways (ko01100); cytosolic DNA-sensing pathway (ko04623); Epstein-Barr virus infection (ko05169); purine metabolism (ko00230); pyrimidine metabolism (ko00240) |
| GB40746 | peptidyl-prolyl cis-trans isomerase FKBP4 | estrogen signaling pathway (ko04915); pathways in cancer (ko05200) |

a; Gene ID, BeeBase gene identifiers of the honey bee genome assembly 4.5^68,69^

b; Gene description based on the National Center for Biotechnology Information^70^, and g:profiler search for cellular component gene ontology terms^71^

c; Biological pathways and KEGG ontology identifiers (ko) identifiers from biological pathways based on KASS search^66^.

**Table S12**. KEGG pathways analysis of the DEGs (up-regulated) between the bees exposed to 1X10^-2^ ng of clothianidin per µl plus *V. destructor* compared to bees exposed to 0 ng of clothianidin per µl (0vs1X10^-2^+Vd).

| **Gene ID^a^** | **Gene description^b^** | **Biological pathway^c^** |
| --- | --- | --- |
| GB47618 | defensin 2 | Toll and Imd signaling pathway (ko04624) |
| GB41338 | venom acid phosphatase | cellular senescence (ko04218) |
| GB52775 | hyaluronoglucosaminidase | metabolic pathways (ko01100); glycosaminglycan degradation (ko00531) |
| GB52056 | insulin-like growth factor 2 mRNA-binding protein 1 | microRNAs in cancer (ko05206) |
| GB50933 | GATA-binding factor A | cGMP-PKG signaling pathway (ko04022); tight junction (ko04530); thyroid hormone signaling pathway (ko04919) |
| GB50627 | putative fatty acil-CoA reductase | cutin, suberine and wax biosynthesis (ko0073); peroxisome (ko04146); longevity regulating pathway-worm (ko04212) |
| GB54097 | malvolio | lysosome (ko04142); ferroptosis (ko04216); mineral absorption (ko04978) |
| GB44548 | glucose dehydrogenase | metabolic pathways (ko01100); glycine, serine and threonine metabolism (ko00260) |
| GB51874 | SLIT-ROBO Rho GTPase-activating protein 1-like | axon guidance (ko04360) |
| GB54226 | unconventional myosin-Ixb | hippo signaling pathway-fly (ko04391) |
| GB40673 | lambda crystallin-like protein | metabolic pathways (ko01100); pentose and glucuronate interconversions (ko00040) |
| GB51188 | lysophospholipid ayltransferase 2 | metabolic pathways (ko01100); biosynthesis of secondary metabolites (ko01110); glycerolipid metabolism (ko00561); glycerophospholipid metabolism (ko00564) |
| GB47059 | protein tramtrack, beta isoform | MAPK signaling pathway-fly (ko04013) |
| GB49376 | F-box/LRR-repeat protein 3-like | circadian rhythm (ko04710) |
| GB46422 | proton-coupled amino acid transporter 1 | autophagy-yeast (ko04138) |
| GB54595 | histone demethylase UTY | transcriptional misregulation in cancer (ko05202) |
| GB51809 | max-binding protein MNT | calcium signaling pathway (ko04020); insulin signlaing pathway (ko04910); glucagon signlaing pathway (ko04922) |

a; Gene ID, BeeBase gene identifiers of the honey bee genome assembly 4.5^68,69^

b; Gene description based on the National Center for Biotechnology Information^70^, and g:profiler search for cellular component gene ontology terms^71^

c; Biological pathways and KEGG ontology identifiers (ko) identifiers from biological pathways based on KASS search^66^.

**Table S13.** KEGG pathways analysis of the DEGs (down-regulated) between the bees exposed to 1X10^-2^ ng of clothianidin per µl plus *V. destructor* compared to bees exposed to 0 ng of clothianidin per µl (0vs1X10^-2^+Vd).

| **Gene ID^a^** | **Gene description^b^** | **Biological pathway^c^** |
| --- | --- | --- |
| GB42053 | epididymal secretory protein E1-like | lysosome (ko04142) |
| GB45495 | heat shock protein 83 | PI3K-Akt signaling pathway (ko04151); necroptosis (ko04217); NOD-like receptor signaling pathway (ko04621); antigen processing and presentation (ko04612); Th17 cell differentiation (ko04659); IL-17 signaling pathway (ko04657); estrogen signaling pathway (ko04915); progesterone-mediated oocyte maturation (ko04914); plant-pathogen interaction (ko04626); pathways in cancer (ko05200); prostate cancer (ko05215); fluid shear stress and atherosclerosis (ko05418) |
| GB50609 | heat shock protein Hsp70Ab-like | spliceosome (ko03040); protein processing in endoplasmic reticulum (ko04141); MAPK signaling pathway (ko04010); endocytosis (ko04144); antigen processing and presentation (ko04612); estrogen signaling pathway (ko04915); longevity regulating pathway-multiple species (ko04213); influenza A (ko05164); Epstein-Barr virus infection (ko05169); toxoplasmosis (ko05145) |
| GB49250 | heme oxygenase | metabolic pathways (ko01100); biosynthesis of secondary metabolites (ko01110); porphyrin and chlorophyll metabolism (ko00860); mineral absorption (ko04978) |
| GB40746 | peptidyl-prolyl cis-trans isomerase FKBP4 | estrogen signaling pathway (ko04915) |
| GB42964 | beta-1,3-glucosyltransferase | other types of O-glycan biosynthesis (ko00514) |
| GB45228 | chondroitin sulfate synthase 2 | metabolic pathways (ko01100); glycosaminoglycan biosynthesis-chondroitin sulfate/dermatan sulfate (ko00532) |
| GB44298 | enoyl-CoA delta isomerase 1, mitochondrial-like | fatty acids degradation (ko00071); legionellosis (ko05134); measles (ko05162) |

a; Gene ID, BeeBase gene identifiers of the honey bee genome assembly 4.5^68,69^

b; Gene description based on the National Center for Biotechnology Information^70^, and g:profiler search for cellular component gene ontology terms^71^

c; Biological pathways and KEGG ontology identifiers (ko) identifiers from biological pathways based on KASS search^66^.
